# A Non-Linear Biostatistical Graphical Modeling of Preventive Actions and Healthcare Factors in Controlling COVID-19 Pandemic

**DOI:** 10.3390/ijerph18094491

**Published:** 2021-04-23

**Authors:** Faruq Abdulla, Zulkar Nain, Md. Karimuzzaman, Md. Moyazzem Hossain, Azizur Rahman

**Affiliations:** 1 Department of Statistics, Faculty of Sciences, Islamic University, Kushtia 7003, Bangladesh; faruqiustat09mnil@gmail.com; 2 Research, Training and Management (RTM) International, Mirpur, Dhaka 1216, Bangladesh; 3 Department of Biotechnology and Genetic Engineering, Faculty of Biological Sciences, Islamic University, Kushtia 7003, Bangladesh; znain.bd@gmail.com; 4 Department of Statistics, Faculty of Mathematical and Physical Sciences, Jahangirnagar University, Savar, Dhaka 1342, Bangladesh; karimuzzaman.statju@gmail.com (M.K.); M.M.Hossain2@newcastle.ac.uk (M.M.H.); 5 School of Mathematics, Statistics & Physics, Newcastle University, Newcastle upon Tyne NE1 7RU, UK; 6 School of Computing and Mathematics, Charles Sturt University, Wagga Wagga, NSW 2650, Australia

**Keywords:** COVID-19 pandemic, social distance, lockdown, quarantine, case fatality rate, life expectancy

## Abstract

Background: With the insurgence of the COVID-19 pandemic, many people died in the past several months, and the situation is ongoing with increasing health, social, and economic panic and vulnerability. As most of the countries relying on different preventive actions to control the outcomes of COVID-19, it is necessary to boost the knowledge about the effectiveness of such actions so that the policymakers take their country-based appropriate actions. This study generates evidence of taking the most impactful actions to combat COVID-19. Objective: In order to generate community-based scientific evidence, this study analyzed the outcome of COVID-19 in response to different control measures, healthcare facilities, life expectancy, and prevalent diseases. Methods: It used more than a hundred countries’ data collected from different databases. We performed a comparative graphical analysis with non-linear correlation estimation using R. Results: The reduction of COVID-19 cases is strongly correlated with the earliness of preventive initiation. The apathy of taking nationwide immediate precaution measures has been identified as one of the critical reasons to make the circumstances worse. There is significant non-linear relationship between COVID-19 case fatality and number of physicians (NCC = 0.22; *p*-value ≤ 0.001), nurses and midwives (NCC = 0.17; *p*-value ≤ 0.001), hospital beds (NCC = 0.20; *p*-value ≤ 0.001), life expectancy of both sexes (NCC = 0.22; *p*-value ≤ 0.001), life expectancy of female (NCC = 0.27; *p*-value ≤ 0.001), and life expectancy of male (NCC = 0.19; *p*-value ≤ 0.001). COVID-19 deaths were found to be reduced with increased medical personnel and hospital beds. Interestingly, no association between the comorbidities and severity of COVID-19 was found excluding asthma, cancer, Alzheimer’s, and smoking. Conclusions: Enhancing healthcare facilities and early imposing the control measures could be valuable to prevent the COVID-19 pandemic. No association between COVID-19 and other comorbidities warranted further investigation at the pathobiological level.

## 1. Introduction

Currently, the world has been experiencing a newly emerged pandemic causing outbreaks and global catastrophes [1,2,3]. The etiological agent of this pandemic is a positive-sense single-stranded RNA virus designated as severe acute respiratory syndrome coronavirus 2 (SARS-CoV-2), while the disease has been named as coronavirus disease of 2019 (COVID-19) [1,4]. Analysis of the COVID-19 patients confirmed multi-dimensional pathophysiological processes including direct viral invasion, dysregulated renin-angiotensin-aldosterone system (RAAS), hypoxia, hyperinflammation, cytokine storm, endotheliopathy, and thrombosis [5]. These pathophysiological processes are associated with various health complications. In critical COVID-19 patients, disseminated intravascular coagulation (DIC) is a severe health problem caused by the occurrence of thromboinflammation leading to fibrin deposition and microvascular thrombosis [5]. The disseminated intravascular coagulation (DIC) is more strongly correlated with COVID-19 [6]. A study of 183 patients reported 71.4% of non-survivors and 0.6% of recovered cases met the criteria for disseminated intravascular coagulation during hospitalization [7,8]. Other pathophysiological mechanisms are associated with a severe form of respiratory illness, including pneumonia, fever, diarrhea, breathing difficulty, lack of smell/test, and lung dysfunctions with 4.5% of global mortality rate [9,10,11]. As of 25 April 2020, COVID-19 has attributed with a total of 2,832,441 confirmed cases and has claimed 197,342 lives worldwide (https://worldometers.info/coronavirus/, accessed on 20 April 2020) [12]. By considering the spread and severity of COVID-19 with the alarming levels of global loss, the world health organization (WHO) has declared the COVID-19 situation as a pandemic and announced an event called public health emergency of international concern (PHEIC) indicating a significant threat to global health security [13,14].

The primary source of the COVID-19 infection is infected patients as its being contagious [10,15]. The rate of severity and recovery from the COVID-19 is involved with the age, biological sex, and other health conditions of the infected patients [11]. Furthermore, asymptomatic patients may play a critical role in the transmission process [16]. Moreover, studies suggest that various comorbidities (i.e., diabetes mellitus, cardiovascular, and respiratory disease) might influence the COVID-19 progression [10,17,18]. Scientists are actively trying to understand the SARS-CoV-2 as well as its pathogenic nature to develop an efficient antiviral drug or vaccine [11,19,20]. During the first wave of COVID-19, there were no effective antiviral drugs or vaccines against COVID-19 [21,22].

The enactment of emergency capacity-building policies and legislation, combined with thoughtful government leadership, has a profound effect on how a national emergency is handled [23]. While pandemic and health policies rely heavily on healthcare and medical professionals, they also require the integration of various organs of society, including citizens, the media, digital health [24], governments at all levels, including e-government [25] and local government [26], as well as a diverse array of organizations and individuals involved in policymaking and implementation. For instance, the media, which includes traditional print and broadcast media, the internet, and social media, plays a critical role because the manner in which news is reported affects people’s behaviors and attitudes [25,27,28], particularly during an epidemic or pandemic.

Moreover, danger, emotion and risk perception, panic, prejudice and discrimination, social norms, community, inequality, and political polarization; on the other hand, fake news and disinformation, persuasion, conspiracy theories, moral decision-making, engagement, trust and enforcement, aligning person and mutual interests, stress, social isolation and interaction, relationship building, and healthy mind-sets all have a significant impact on how individuals cope with pandemics [28,29,30,31]. As a result of implementing precautionary measures, daily life has a plethora of obstacles, including work life, social life, psychological problems, tourism, economic growth, and individual financial potentiality [28,29,31,32]. For instance, the healthcare professional faces a plethora of problems, resulting in inadequate precautionary practice [30,33,34]. There is significant relationship between several actions and air quality, clean beaches, and environmental noise; however, the rise in waste level and curtailment in recycling may impact future environment [35].

For now, therefore, our best interest lies in subduing the symptoms/complications with available medications and controlling the viral spread through different control measures at national and global scales [21,36]. In response to COVID-19, many countries have already taken such initiatives, such as epicenter lockdown, identifying the carrier and patients, maintaining social distances, restriction on visa and traveling, limiting public gathering, extending medical facilities, wearing masks, use of ventilation, and so on [13,37]. However, the costs of these measures for a prolonged period would be very high, resulting in an unparalleled socio-economic loss in the history of humanity. Besides, domestic violence and other psychological disorders may have increased due to the prolonged period of quarantine [38]. The actual consequences of COVID-19 remain to be observed. Some scientists also suggested developing herd immunity, an epidemiological term describing a sufficient number of immune individuals, through mass infection/vaccination [39]. In support, some pointed out that social isolation/disconnection could heighten the risk of prevailing health conditions and mental problems [40,41,42].

Conversely, some researchers are opposed to their notion regarding herd immunity. In their opinion, developing herd immunity without vaccination will result in a catastrophic outcome since the infections cover 70% of all populations with a 0.25–3% case fatality rate [43]. Acquiring herd immunity will not be effective against SARS-CoV-2 due to the continuous evolution of its genome [44]. Therefore, it is a high time to evaluate the effectiveness of the preventive measure in containing the situation and to value if they evaluate outweigh the costs involved. To the best of our knowledge, however, no such studies have done this yet.

In this study, we used different statistical methods to understand the relationships between the control measures and the country-wise infection index. The association of different comorbidities with the severity of the COVID-19 disease was also evaluated in this limited scope. We aim to find the crucial factors in containing the situation more rationally and suggest significant evidence-based interventions to the law enforcement personnel and policymakers accordingly. Also, this study will shed light on the essential aspects that if specific comorbidities could have worsened the severity of COVID-19 and vice-versa.

## 2. Materials and Methods

### 2.1. Data and Variable

To explore the association of preventive approaches and the COVID-19 outcome, we used several secondary databases for information regarding country-wise control measures—i.e., epicenter lockdown, restriction in traveling and public services, healthcare measures, and many more. In addition, life expectancy, prevalence, and death rates in prevalent diseases and risk factors were considered for each country. Herein, country-based life expectancy and the COVID-19 outcome (i.e., confirmed cases and deaths) data has been curated from the Worldometer (https://worldometers.info/coronavirus, accessed on 20 April 2020) [12]. Worldometer is a website owned and operated by a data company Dadax which generates real-time data and statistics on the diverse topic including world population, COVID-19, and health. For healthcare measures undertaken in different countries, we employed the Humanitarian Data Exchange (https://data.humdata.org/, accessed on 20 April 2020) [45]. The Humanitarian Data Exchange (HDX) is an open platform managed by OCHA’s Centre for Humanitarian Data and intends to make humanitarian data easy to find and use for analysis and sharing data across crises and organizations. These measures have been analyzed to check their effectiveness on the outbreak of COVID-19 by a comparative study between countries with high and low case fatality rates. Analyzing procedure involves the days calculated from the first case confirmation of a distinct nation. Also, the number of physicians, nurses, and hospital beds was obtained from the Our World in Data website (https://ourworldindata.org/, accessed on 20 April 2020) [46]. Our World in Data (OWID) is a scientific online publication under the project of the Global Change Data Lab published by the research team is based at the University of Oxford that focuses on large global problems such as poverty, disease, hunger, climate change, war, existential risks, and inequality.

Furthermore, we evaluated the association of the number of physicians, nurses and midwives, hospital beds, and life expectancy with the COVID-19 case fatality rate. Later on, the non-linear relationships between COVID-19 case fatality rate and healthcare resources were measured with their respective adjusted *p*-value. Then, we assessed the correlation of fatal diseases with the progression of COVID-19 complications by using prevalence and death rates in several prevalent diseases and risk factors. The data regarding country-wise disease death rates and life expectancy were collected from World Life Expectancy (https://worldlifeexpectancy.com/, accessed on 20 April 2020) [47] while prevalence rates and smoking-related statistics obtained from Our World in Data (https://ourworldindata.org/, accessed on 20 April 2020) [46]. World Life Expectancy is one of the largest global health and life expectancy databases in the world. All the data used in this study have collected on 20 April 2020.

The comparative analysis among the selected countries was done using the R graphics package. While a contemporary heuristic approach was utilized for estimating the various non-linear associations between significant variables considered in this study with the R nlcor-package [48]. All analyses for this research were completed in R Software (Version 3.6.1, R Foundation for Statistical Computing, Vienna, Austria).

### 2.2. Methods

The concept nonlinear correlation coefficient was developed by Wang et al. (2005) [49] and they demonstrated that the mutual information carried out by the rank sequences is a good measure of nonlinear correlation. Let, X and Y be the two discrete variables, they are first resorted in ascending order and placed into b ranks with first $\frac{N}{b}$ samples in the first rank, the second $\frac{N}{b}$ samples in the second rank and so on. Second, the sample pairs $\left(x_{i},y_{i}\right);\;i=1,2,\ldots N$ are placed into b×b rank grids by comparing the sample pairs to the rank sequences of X and Y. The revised joint entropy of the two variables X and Y is defined as

(1) $$H^{r}\left(X,Y\right)=-\sum_{i=1}^{b}\sum_{j=1}^{b}\frac{n_{ij}}{N}\log_{b}\left(\frac{n_{ij}}{N}\right)$$

where, $n_{ij}$ is the number of samples distributed in the (*ij*)th rank grid and N is the total number of pairs.

Then the nonlinear correlation coefficient is defined as

(2) $$NCC\left(X,Y\right)=H^{r}\left(X\right)+H^{r}\left(Y\right)-H^{r}\left(X,Y\right)$$

where, $H^{r}\left(X\right)$ is the revised entropy of the variable X and defined as

(3) $$H^{r}\left(X\right)=-\sum_{i=1}^{b}\frac{n_{i}}{N}\log_{b}\left(\frac{n_{i}}{N}\right)$$

Since the number of samples distributed into each rank of X and Y is invariant so the nonlinear correlation coefficient (2) can be written as

(4) $$NCC\left(X,Y\right)=2+\sum_{i=1}^{b^{2}}\frac{n_{ij}}{N}\log_{b}\left(\frac{n_{ij}}{N}\right)$$

## 3. Results

### 3.1. COVID-19 and National Preventive Action

**Figure 1:**
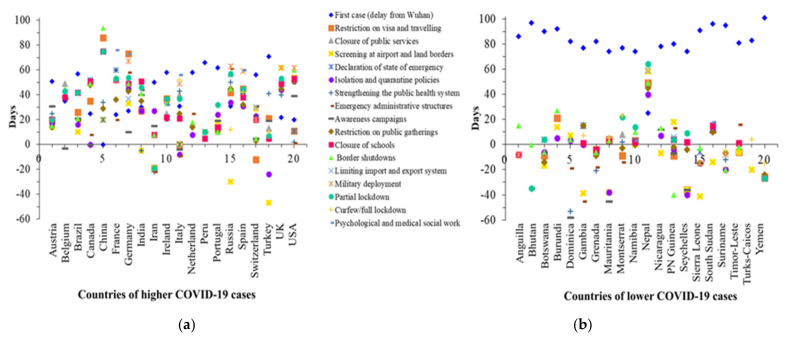
Comparative analysis of the healthcare measures undertaken by the government for top 20 high (**a**) and top 20 low (**b**) case-death countries. The considered measures can be categorized as public health measures (screening at airport and land borders, isolation and quarantine policies, strengthening the public health system, awareness campaigns, psychological, and medical social work), social distancing (closure of public services, restriction on public gatherings, closure of schools), governance and socio-economic measures (declaration of state of emergency, emergency administrative structures, limiting import and export system, military deployment), movement restrictions (restriction on visa and travelling, border shutdowns, curfew), and lockdown (partial lockdown, full lockdown).

The top 20 high case-death countries are financially developed and their healthcare systems are better than the top 20 low case-death countries, but the high case-death countries were unable to tackle the boosting trend of morbidity and mortality properly. One of the principal focuses of this research is to observe the effectiveness of the different preventive action plans taken by the government to control the outbreak of COVID-19. A total of 40 countries (i.e., top 20 high and top 20 low case-death countries) data have analyzed to discover the intrinsic grounds of their current conditions, as illustrated in Figure 1. In most countries among the 20 profoundly affected countries, the visa restriction policy has been employed after a couple of weeks of the first COVID-19 case report, except for Iran and Switzerland. Congruently, many of the countries showed their apathy to prioritize the screening in airports and borders. In contrast, the starting of declaring a state of emergency has been seen distinctly by the top burdening. Figure 1 also reveals that excluding the USA, Iran, and India, all the top burdening nations show aloofness in the solidification of the public health structure. Among the top burdening nations USA, Italy, Iran, and Canada seem to take immediate action to stimulate emergency administrative structure compatibly, when the first confirmed cases of the disease identified. It is a scandalous finding that most of the top burdening countries introduce isolation and quarantine policies a minimum of 16 days later except few countries. Compatibly, limiting the public gathering through closing the schools, border, and public service is a splendid feature of handling the epidemic, and our findings are consistent with the findings of other research [50,51,52,53].

Additionally, reliable decisions such as military deployment to support the law enforcement agencies, partial lockdown, and curfew to limit public gathering have been implemented latterly by most of the country—excluding Russia, Iran, and Turkey. Our findings also suggest that the scientific education-based public awareness, intense healthcare systems preparation, limitation of large-scale public gathering, isolation, social distancing, testing, lockdown, and other preventive measures to prevent the pandemic were effective in many societies (Figure 1). These findings are supported by several studies that were applicable to the previous epidemic [13,52,54,55,56,57,58,59].

On the contrary, the country with the least deaths and infections find their first infected person at a minimum of 74 days later than its origin in Wuhan, excluding Nepal. Almost all countries found a substantial window of time to take immediate action and preventive measures for individuals. Most of them starting visa restrictions, administrative emergency, limiting public gatherings, isolation policies, and partial lockdown before the identification of the first case. Some studies reported that early implemented lockdown will be more effective to lessen the burden of morbidity and mortality from COVID-19 [23,60].

Nevertheless, only a few countries implemented full lockdown as well as a testing policy in rapid response. Conversely, Nepal implemented almost every preventive measure on a delayed timeline, but lately, there is a shortage of COVID-19 patients, which may indicate the successful implication of testing policy as they have done the highest number of tests. Taking account of steps implemented by different countries, this study marks a few countries as high risk based on the analysis of the preventive measure of the top 20 in terms of virus burdening. Among the least affected countries Gambia, Nicaragua, Burundi, Namibia, and Nepal are in a state of danger as they have taken more of the preventive measures than others (Figure 1). These marked countries do not implement the testing policy correctly though, and three of them even do not implement the full lockdown policy also, which increases their chance to become a top burdening country. Furthermore, an interesting finding of our research is that almost every country began its awareness campaign more swiftly relative to the first patient being identified (Figure 1). Still, they are at the peak of affected countries, which makes the robust validation of claiming that the scientific evidence of infection positively correlates to lower public awareness, and it is consistent with findings from a recent study [59]. Interestingly, from the results, it is seen that there is a significant difference between the top 20 high and top 20 low case-death countries. In the lower cases reported countries, most of the preventive actions—especially restriction on movements, screening at airports and land borders, and lockdown—were taken quickly—i.e., on or before 20 days after identified the first case in China. However, the countries whose COVID-19 case counts are higher took preventive measures after a longer period—i.e., they took most of their actions after 20 days (Figure 1).

### 3.2. Healthcare Resources in COVID-19

**Figure 2:**
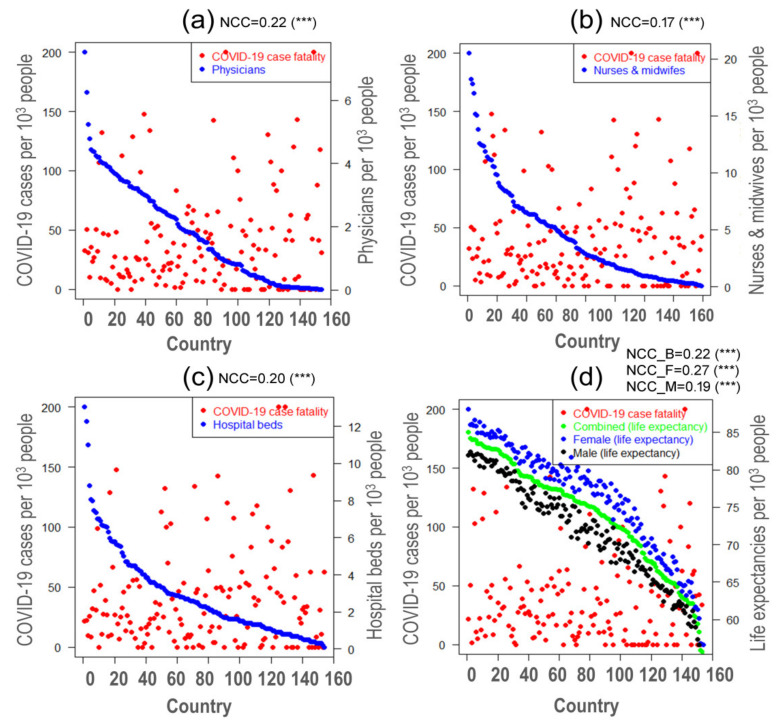
Relationship among COVID-19 case fatality, healthcare resources, and life expectancy where (**a**–**c**) represent the association between COVID-19 case fatality and the number of physicians, nurses and midwives, and hospital beds, respectively while (**d**) indicates the relationship between COVID-19 case fatality and life expectancy by gender for all 154 countries where COVID-19 infection was identified on 20 April 2020; *** represent significant at 0.1%. Note: The country list corresponding to the index in the horizontal axis of all figures are presented in Table S1.

The correlation between COVID-19 case fatality and health service parameters has been evaluated to assess their effect on the current pandemic as shown in Figure 2a–c where the country list corresponding to the index in the horizontal axis of all figures are presented in Table S1. Herein, all the resources have taken with an inclusion of the calculation of per 1000 peoples. It has been speculated that the COVID-19 case fatality is strongly associated with these resources. We found 0.22, 0.17, and 0.20 as the measurement of a non-linear relationship between COVID-19 case fatality and the number of physicians, nurses and midwives, and hospital beds and these relationships are highly significant with a *p*-value less than 0.001. As expected, the COVID-19 case fatality is high in those countries that have a lower number of physicians, hospital beds, and nurses and midwives. This observation is plausible since more doctors could treat more patients while fast healthcare could have provided more nurses and hospital beds. In a recent study, Ji et al. (2020) reported that the COVID-19 mortality correlates with the healthcare resources in China; and they concluded that insufficient healthcare resources increase the COVID-19 mortality [61]. In a previous study, Farahani et al. (2016) revealed that death from HIV would reduce by 27%, 28%, and 9% if doctors could have double, nurses can increase from 20 to 25, and five hospital beds per 10,000 people, respectively [62]. The shortage of the selected health system resources results in the delay of care and affects the quality of care that eventually leads to more critical patients and case fatality [63]. For instance, Kruk et al. (2018) showed that 5 million excess deaths occurred due to the lack of quality healthcare in 137 low- and middle-income countries in 2016 [64]. Therefore, an increasing number of physicians, hospital beds, and nurses and midwives can significantly decrease the number of deaths from the COVID-19 pandemic.

### 3.3. Life Expectancy and COVID-19 Case Fatality

Aging is a complex cellular and molecular processes which may lead to functional and structural changes in the immune system [65]. In addition, weak immunity is listed as one of the major risk factors in many chronic disorders including infections, cancer, cardiovascular, and neurodegenerative diseases [66,67]. Since COVID-19 is a viral disease, any changes in the immune system might influence the COVID-19 and related complications [68,69]. Therefore, we investigated the relationship between COVID-19 case fatality and the human lifespan. Due to the unavailability of the age-specific information of the COVID-19 cases and deaths, the life expectancy was as it represents the percentage of older people living in a particular country.

Figure 2d illustrates the relationship between life expectancy and COVID-19 case fatality rate (CFR) per 1000 cases. Moreover, the measures of non-linear correlations of COVID-19 case fatality with a life expectancy of both sexes, female, and male were found as 0.22, 0.27, and 0.19 with a *p*-value less than 0.001. It is explicitly seen that the CFR of COVID-19 was substantially higher in countries with the life expectancy ≤80 years for both sexes but the CFR was substantially lower in countries with the life expectancy >80 years. As of 29 June 2020, WHO published a report on focusing Bangladeshi data and it has pointed out that only 3.9% of males and 1.2% of females had the age more than 80 years among the total deaths [70]. This observation is consistent with the previously thought that the CFR of COVID-19 would be higher in people older than 70 years [71]. However, in that study, no highest age limit was mentioned but in our study, the highest age limit was identified as 80 years. The explanation of these findings could lie in their diet and lifestyle. In countries with high lifespan, people tend to have healthy food habits. For instance, a quality diet has been linked to the longevity of Japanese people [72]. Diet can positively influence the overall health and immunity that eventually lead to a long lifespan [73,74]. In a human study, for example, increasing uptake of vitamin E was found to reduce the risk of respiratory infections by 35%, especially in the upper respiratory tract (38%) which COVID-19 initially affects [75]. Thus, our findings may provide an urgent basis to study the effect of COVID-19 on aging or vice-versa.

### 3.4. Association between COVID-19 and Comorbidities

**Figure 3:**
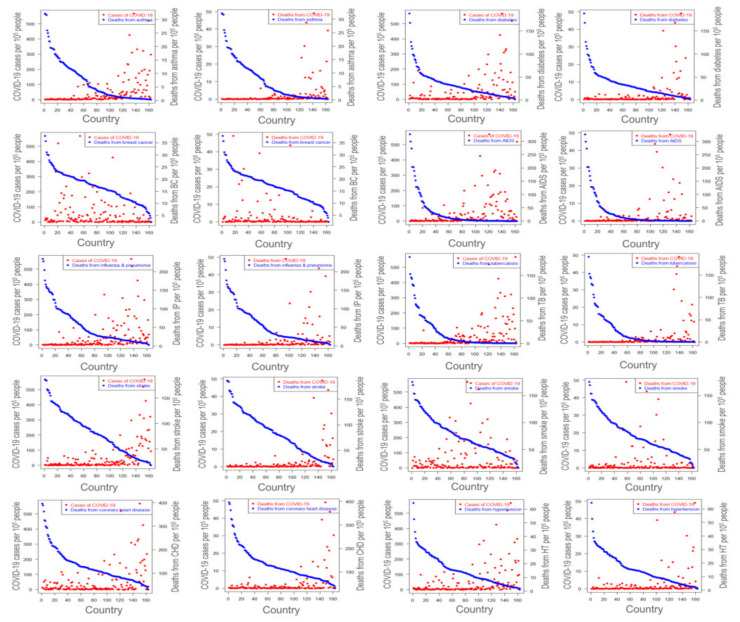
Association between COVID-19 cases and case fatality and some selected comorbidities death rates for all 163 countries where COVID-19 infection was identified on 20 April 2020. Note: Comorbidity variables considered here are asthma, diabetics, breast cancer (BC), AIDS, influenza & pneumonia (IP), tuberculosis, stroke, smoke, coronary heart disease (CHD), and hypertension. Each pair of graphs from left to right with a selected comorbidity variable are showing COVID-19 confirmed cases and deaths respectively. The country list corresponding to the index in the horizontal axis of all figures are presented in Table S2.

Comorbidity is the prevailing conditions that can accelerate the progression and severity of certain diseases [76]. For instance, several studies have reported that cardiovascular diseases, hypertension, and diabetes can increase the severity of COVID-19 [18,77]. Hypothetically, there would be a positive correlation between the COVID-19 cases and these health conditions if they are associated. Interestingly, our study reflects the opposite scenario in most cases except for a few, as shown in Figure 3; the country lists corresponding to the index in the horizontal axis of all figures are presented in Table S2. Notably, countries with higher death rates from asthma, cardiovascular diseases, hypertension, and diabetes showed a significantly lower amount of COVID-19 cases, which is unlike what we previously assumed. The findings reveal that both the number of infected cases and deaths from COVID-19 is high among some countries whose number of death due to breast cancer and Alzheimer’s are also high. However, an adverse scenario is observed for the case of asthma, coronary heart disease, diabetes. It is very difficult to conclude about the association of deaths from diarrhea, and AIDS with the number of deaths due to COVID-19 (Figure 3). Besides, people with underlying health conditions recommended staying at home, and hopefully, they did [78].

**Figure 4:**
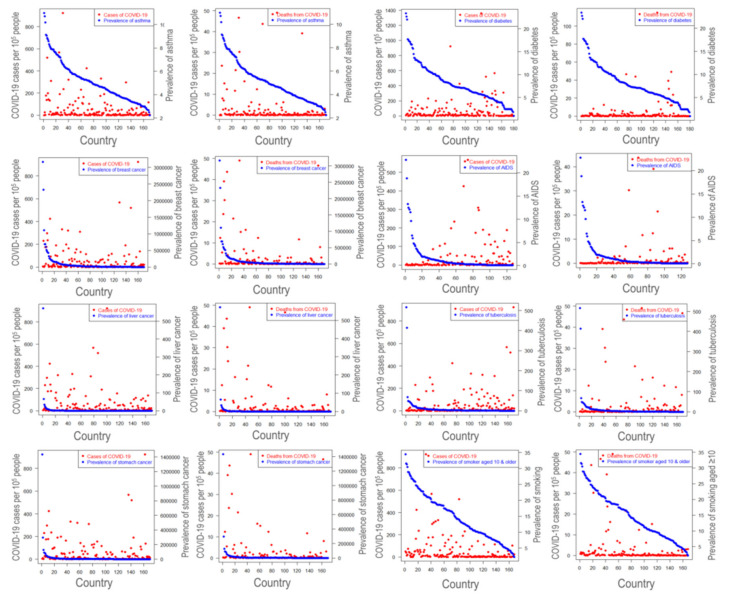
Association between COVID-19 cases and case fatality and some selected comorbidities prevalence rates where COVID-19 infection was identified on 20 April 2020. Note: Comorbidity variables considered here are asthma (170 countries), diabetics (180 countries), breast cancer (BC) (170 countries), AIDS (127 countries), liver cancer (LC) (170 countries), tuberculosis (170 countries), stomach cancer (SC) (170 countries), and smoking (168 countries). Each pair of graphs from left to right with a selected comorbidity variable are showing COVID-19 confirmed cases and deaths respectively. The country list corresponding to the index in the horizontal axis of all figures are presented in Table S3.

People in countries where they tend to develop these disorders could be more careful and have treated with improved health services. These altogether may lead to an unusual scenario we observed in this study. However, breast cancer, leukemia, smoking, lung cancer, and Alzheimer’s have significantly affected the COVID-19 (Figure 3 and Figure S1, and Table S4). Most importantly, the observation was quite similar when analyzed with the country-wise prevalence of several diseases. For example, countries with highly prevalent diabetes, AIDS, tuberculosis, breast cancer, and smoking showed a lower amount of SARS-CoV-2 infection (Figure 4 and Table S3). In the case of asthma, however, COVID-19 cases were higher in prevalent asthma countries, which are unlike the countries with higher death cases from asthma. More specifically, based upon the data considered in this study, it is seen that the prevalence of asthma, breast cancer, smoking behaviors are positively associated with deaths due to COVID-19 in some countries. Moreover, the prevalence of diabetes is connected with COVID-19 deaths to some extent in a few countries. The findings also reveal that there is no potential association between the prevalence of AIDS, liver cancer, stomach cancer, malaria, tuberculosis with both cases and the number of deaths due to COVID-19 in almost all countries considered in this study (Figure 4).

## 4. Conclusions

Based on the individual national risk, the immediate action of response, preparation, and readiness is an obvious step for countries that belongs to 4Cs (no cases, first cases, first clusters, and community transmission and spread). However, this study involves the scrutiny of national preventive actions, healthcare resources, life expectancy, and comorbidities towards the COVID-19 pandemic. Quite a few associations, correlations, as well as comparisons, have been used throughout the investigations to find the relevant insights about the epidemic. The analyses of the top 20 burdening, as well as the least affected countries, have roughly identified the reasons along with the implemented national healthcare measures. Those nations who have shown apathy in taking immediate healthcare actions after the first identified case find their circumstances more dreadful. Similar lethargy in taking immediate healthcare action is also seen in some least burdening countries and those marked as at risk. Moreover, there is an acute emergency of taking immediate healthcare actions to control the pandemic as most of the least burdening countries found the benefits. The evidence of the study validates that the increasing number of doctors, hospital beds, and nurses will result in a decline in the number of deaths from COVID-19. A stimulating finding is that the countries having higher life expectancy appear to have radically lower fatality rates in the epidemic. Surprisingly, the results depict that the countries with higher death rates from asthma, cardiovascular diseases, hypertension, and diabetes exhibited a significantly lower amount of COVID-19 cases, which is more interesting as well as violates the pre assumptions.

Finally, the authors suggest that the scientific education-based public awareness, intense healthcare systems preparation, limitation of large-scale public gathering, isolation, social distancing, testing, lockdown, and other preventive measures are valuable to prevent the current COVID-19 pandemic. Also, we expect that the findings could be helpful for policymakers to take action in controlling the future pandemic. Furthermore, it is realized that non-pharmaceutical interventions are more important and necessary to prevent the ongoing pandemic among countries whose healthcare facilities are not up to standard and experiencing a higher rate of comorbidities. Moreover, developed countries may increase the medical staff, hospital beds, and ventilation along with non-pharmaceutical interventions to combat COVID-19.

There are some limitations of this study. The authors feel that all associated factors were not considered due to the unavailability of the appropriate data for all countries included in this study. This paper aimed to find the association between COVID-19 outcomes and different actions taken by different countries to prevent the spread of this virus. The PCR testing facilities vary from developed countries to developing countries and perhaps it has some impact on the number of infected cases; however, this is not the focus of the current study. Moreover, there is no database for the actual age distribution with cases and death by countries. Therefore, we consider the life expectancy of the selected countries. Furthermore, the overall healthcare infrastructure and the economic power of a country play a key role to control as well as manage the incidences of COVID-19, however, the authors found that information related to this issue is available only for some countries. Since the relevant data is not available for all countries considered in this study, we were unable to incorporate it in this study. The authors think that this may be an interesting topic for further study.

## Data Availability

The full data set is available either upon request to the corresponding author or from the mentioned secondary data sources.

## Supplementary Materials

The following are available online at https://www.mdpi.com/article/10.3390/ijerph18094491/s1, Figure S1: Association between COVID-19 cases and case fatality and some selected comorbidities death rates for all 163 countries where COVID-19 infection was identified on 20 April 2020. Note: Comorbidity variables considered here are Alzheimer’s, stomach cancer, liver cancer, lung cancer, leukemia, kidney disease, inflammatory heart disease, and rheumatic heart disease. In each pair of graphs from left to right with a selected comorbidity variables are showing COVID-19 confirmed cases and deaths respectively. The country lists corresponding to the index in the horizontal axis of all figures are presented in Table S4; Table S1: Country list corresponding to the index in the horizontal axis of Figure 2; Table S2: Country list corresponding to the index in the horizontal axis of Figure 3; Table S3: Country list corresponding to the index in the horizontal axis of Figure 4. Table S4: Country list corresponding to the index in the horizontal axis of Figure S1.
