# Supplementary material for: A Non-Linear Biostatistical Graphical Modeling of Preventive Actions and Healthcare Factors in Controlling COVID-19 Pandemic
Source: Int J Environ Res Public Health. 2021 Apr 23;18(9):4491. doi: 10.3390/ijerph18094491 (PMC8122857; doi:10.3390/ijerph18094491)
Supplement: Supplementary file 1 [file ijerph-18-04491-s001.zip › ijerph-1158533-supplementary.pdf]

Supplementary File

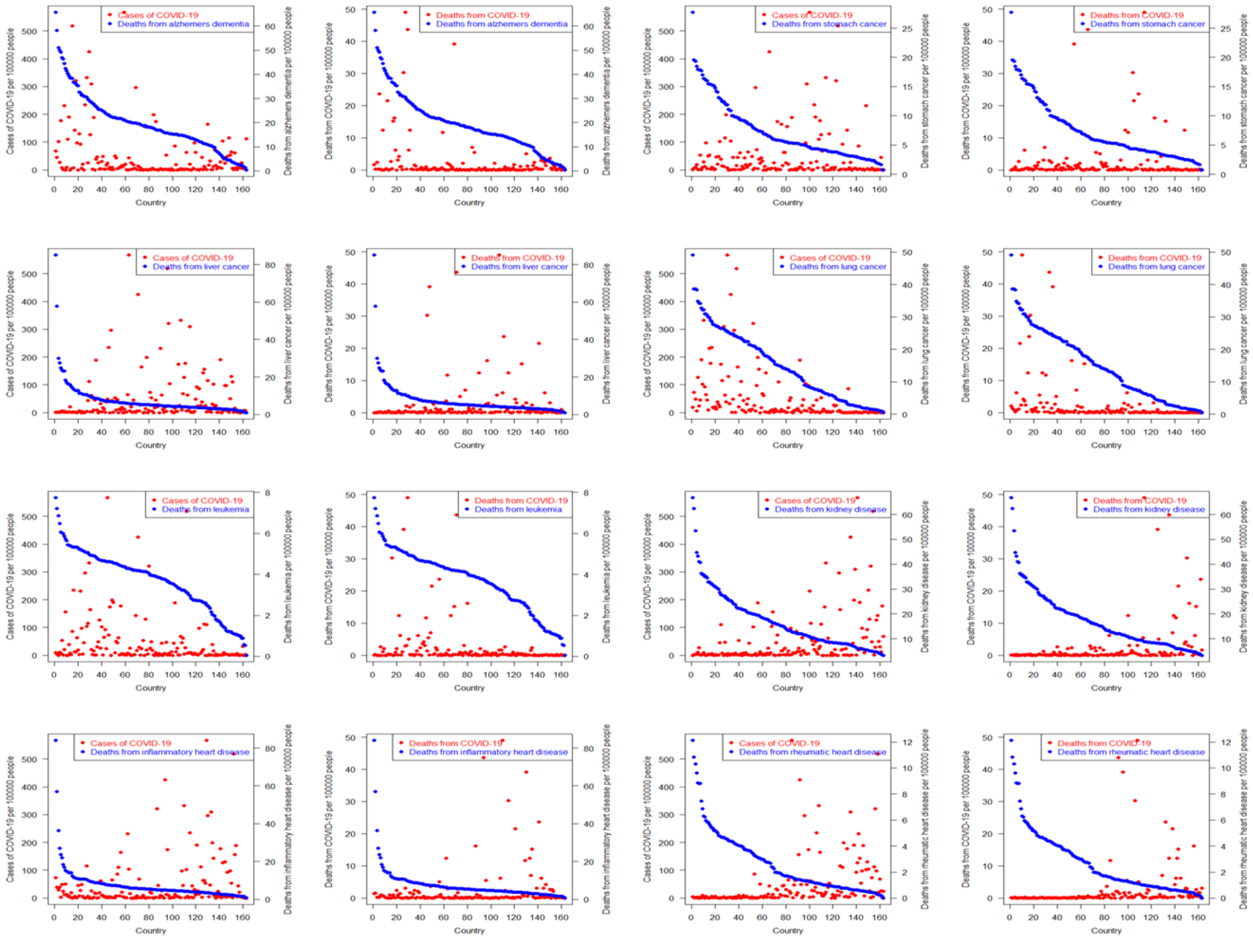

Figure S1. Association between COVID-19 cases and case fatality and some selected comorbidities death rates for all 163 countries where COVID-19 infection was identified on April 20, 2020.[*Note*: Comorbidity variables considered here are alzheimer dementia, stomach cancer, liver cancer, lung cancer, leukemia, kidney disease, inflammatory heart disease, and rheumatic heart disease. In each pair of graphs from left to right with a selected comorbidity variables are showing COVID-19 confirmed cases and deaths respectively. The country list corresponding to the index in the horizontal axis of all figures are presented in Table S1].

Table S1. Country list corresponding to the index in the horizontal axis of Figure 2.

Physician		Nurses and Midwives		Hospital Bed		Life Expectancy	
**Country**	**Index**	**Country**	**Index**	**Country**	**Index**	**Country**	**Index**
Cuba	1	Switzerland	1	Japan	1	Japan	1
Greece	2	Norway	2	South Korea	2	Switzerland	2
Austria	3	Denmark	3	Belarus	3	Singapore	3
Georgia	4	Iceland	4	Ukraine	4	Italy	4
Portugal	5	Finland	5	Russia	5	Spain	5
Norway	6	Germany	6	Germany	6	Australia	6
Lithuania	7	Australia	7	Bulgaria	7	Iceland	7
Switzerland	8	Uzbekistan	8	Austria	8	South Korea	8
Germany	9	Ireland	9	Hungary	9	Israel	9
Sweden	10	Luxembourg	10	Mongolia	10	Sweden	10
Belarus	11	Sweden	11	Romania	11	France	11
Italy	12	Belarus	12	Kazakhstan	12	Malta	12
Bulgaria	13	Japan	13	Czech Republic	13	Canada	13
Russia	14	New Zealand	14	Poland	14	Norway	14
Malta	15	Belgium	15	Lithuania	15	Ireland	15
Argentina	16	France	16	Gabon	16	New Zealand	16
Spain	17	Netherlands	17	France	17	Greece	17
Iceland	18	United States	18	Slovakia	18	Luxembourg	18
Uruguay	19	Canada	19	Barbados	19	Netherlands	19
Czech Republic	20	Malta	20	Moldova	20	Portugal	20
Denmark	21	Slovenia	21	Belgium	21	Finland	21
Maldives	22	Russia	22	Serbia	22	Belgium	22
Israel	23	Kazakhstan	23	Latvia	23	Austria	23
Australia	24	United Kingdom	24	Croatia	24	Germany	24
Netherlands	25	Czech Republic	25	Cuba	25	Slovenia	25
Slovakia	26	Austria	26	Argentina	26	United Kingdom	26
Estonia	27	Maldives	27	Azerbaijan	27	Cyprus	27
Jordan	28	Lithuania	28	Estonia	28	Denmark	28
Azerbaijan	29	Cuba	29	Switzerland	29	Costa Rica	29
Kazakhstan	30	Brazil	30	Luxembourg	30	Chile	30
Mongolia	31	Singapore	31	Slovenia	31	Qatar	31
France	32	Kuwait	32	Kyrgyzstan	32	Maldives	32
Latvia	33	Ukraine	33	Malta	33	Czech Republic	33
Moldova	34	Libya	34	China	34	Barbados	34
Finland	35	South Korea	35	Maldives	35	Poland	35
Croatia	36	Azerbaijan	36	Greece	36	Lebanon	36
Hungary	37	Hungary	37	Armenia	37	Cuba	37
New Zealand	38	Croatia	38	Papua New Guinea	38	Estonia	38

Belgium	39	Brunei	39	Uzbekistan	39	United States	39
Ukraine	40	Romania	40	Montenegro	40	Panama	40
Ireland	41	Portugal	41	Australia	41	Croatia	41
Luxembourg	42	Estonia	42	Libya	42	Albania	42
United Kingdom	43	Kyrgyzstan	43	Norway	43	Oman	43
Slovenia	44	Slovakia	44	Sri Lanka	44	United Arab Emirates	44
Armenia	45	Bosnia and Herzegovina	45	Seychelles	45	Turkey	45
Romania	46	Montenegro	46	Bosnia and Herzegovina	46	Uruguay	46
Kuwait	47	Italy	47	Mauritius	47	Slovakia	47
United States	48	Qatar	48	Cyprus	48	Bosnia and Herzegovina	48
Saudi Arabia	49	Poland	49	Portugal	49	Colombia	49
Canada	50	Uruguay	50	Netherlands	50	Thailand	50
Cyprus	51	Armenia	51	Finland	51	Bahrain	51
Serbia	52	Spain	52	Italy	52	Ecuador	52
Uzbekistan	53	Bulgaria	53	Suriname	53	Sri Lanka	53
Lebanon	54	Suriname	54	Trinidad and Tobago	54	Algeria	54
Japan	55	South Africa	55	Israel	55	China	55
Montenegro	56	Saudi Arabia	56	Spain	56	Peru	56
South Korea	57	Israel	57	Ireland	57	Morocco	57
Poland	58	Latvia	58	Iceland	58	Montenegro	58
Singapore	59	Barbados	59	Lebanon	59	Tunisia	59
Mexico	60	Serbia	60	Sao Tome and Principe	60	Iran	60
Libya	61	Moldova	61	Albania	61	Hungary	61
Mauritius	62	Seychelles	62	Turkey	62	Argentina	62
Qatar	63	Argentina	63	Uruguay	63	Saint Lucia	63
Oman	64	Oman	64	United States	64	Malaysia	64
Bosnia and Herzegovina	65	Malaysia	65	Brunei	65	Brazil	65
Kyrgyzstan	66	Cyprus	66	Saudi Arabia	66	Romania	66
Brazil	67	Mongolia	67	Namibia	67	Serbia	67
Trinidad and Tobago	68	Georgia	68	New Zealand	68	Lithuania	68
Colombia	69	Albania	69	Vietnam	69	Brunei	69
China	70	Greece	70	Georgia	70	Kuwait	70
Barbados	71	Mauritius	71	United Kingdom	71	Vietnam	71
Turkey	72	Trinidad and Tobago	72	Canada	72	Latvia	72
Brunei	73	Jordan	73	Denmark	73	Saudi Arabia	73
Ecuador	74	United Arab Emirates	74	Singapore	74	Armenia	74
El Salvador	75	Fiji	75	South Africa	75	Mauritius	75
Panama	76	Gabon	76	Panama	76	Bulgaria	76
United Arab Emirates	77	Sri Lanka	77	Tunisia	77	Mexico	77
Malaysia	78	Namibia	78	Fiji	78	Nicaragua	78

Dominican Republic	79	Botswana	79	Sweden	79	Belarus	79
Iran	80	Mexico	80	Brazil	80	Belize	80
Tunisia	81	Tunisia	81	Chile	81	Guatemala	81
Albania	82	Turkey	82	Thailand	82	Jordan	82
Paraguay	83	Lebanon	83	Bahrain	83	Jamaica	83
Algeria	84	Bahrain	84	Kuwait	84	Dominican Republic	84
Costa Rica	85	China	85	Zambia	85	Paraguay	85
Peru	86	Panama	86	Algeria	86	Georgia	86
Philippines	87	Thailand	87	Malaysia	87	El Salvador	87
Chile	88	India	88	Botswana	88	Trinidad and Tobago	88
Seychelles	89	Ecuador	89	Colombia	89	Kazakhstan	89
Pakistan	90	Sao Tome and Principe	90	Jamaica	90	Seychelles	90
Bahrain	91	Nepal	91	Bhutan	91	Bangladesh	91
Nicaragua	92	Saint Lucia	92	Zimbabwe	92	Libya	92
Guatemala	93	Belize	93	Oman	93	Azerbaijan	93
Sri Lanka	94	Algeria	94	Peru	94	Russia	94
Iraq	95	Iraq	95	Dominican Republic	95	Bhutan	95
Fiji	96	Jamaica	96	Egypt	96	Egypt	96
Belize	97	Gambia	97	Guyana	97	Ukraine	97
Vietnam	98	Nigeria	98	Rwanda	98	Bolivia	98
South Africa	99	Kenya	99	Congo	99	Indonesia	99
Suriname	100	Iran	100	Ecuador	100	Moldova	100
Egypt	101	Bhutan	101	Iran	101	Suriname	101
India	102	Peru	102	Laos	102	Uzbekistan	102
Morocco	103	Angola	103	Jordan	103	Kyrgyzstan	103
Nepal	104	Vietnam	104	Iraq	104	Nepal	104
Myanmar	105	Egypt	105	Djibouti	105	Philippines	105
Sao Tome and Principe	106	Nicaragua	106	Kenya	106	Iraq	106
Laos	107	Dominican Republic	107	Mexico	107	Sao Tome and Principe	107
Bolivia	108	Indonesia	108	Saint Lucia	108	Cambodia	108
Jamaica	109	Zimbabwe	109	Belize	109	Mongolia	109
Bangladesh	110	Sudan	110	Paraguay	110	India	110
Thailand	111	Colombia	111	El Salvador	111	Guyana	111
Gabon	112	Paraguay	112	Malawi	112	Rwanda	112
Nigeria	113	Bolivia	113	Cameroon	113	Botswana	113
Botswana	114	Laos	114	Qatar	114	Laos	114
Bhutan	115	Cambodia	115	United Arab Emirates	115	Senegal	115
Namibia	116	Myanmar	116	Costa Rica	116	Madagascar	116
Yemen	117	Ghana	117	Morocco	117	Fiji	117
Afghanistan	118	Zambia	118	Bolivia	118	Djibouti	118

Sudan	119	Morocco	119	Gambia	119	Pakistan	119
Djibouti	120	Guatemala	120	Indonesia	120	Myanmar	120
Guyana	121	Rwanda	121	Philippines	121	Eritrea	121
Kenya	122	Congo	122	Guinea-Bissau	122	Kenya	122
Indonesia	123	Costa Rica	123	Nicaragua	123	Gabon	123
Benin	124	Yemen	124	Myanmar	124	Yemen	124
Angola	125	Mauritania	125	Ghana	125	Tanzania	125
Cambodia	126	Uganda	126	Somalia	126	Sudan	126
Madagascar	127	Burkina Faso	127	Bangladesh	127	Afghanistan	127
Gambia	128	Eritrea	128	Cambodia	128	Malawi	128
Saint Lucia	129	Benin	129	Sudan	129	Mauritania	129
Ghana	130	Djibouti	130	Liberia	130	Papua New Guinea	130
Congo	131	Guinea-Bissau	131	Burundi	131	Congo	131
Uganda	132	Papua New Guinea	132	Angola	132	Liberia	132
Zambia	133	Guyana	133	Eritrea	133	Ghana	133
Mali	134	Cameroon	134	Yemen	134	South Africa	134
Cameroon	135	Pakistan	135	Tanzania	135	Namibia	135
Zimbabwe	136	Liberia	136	Mozambique	136	Zambia	136
Guinea	137	Mali	137	Togo	137	Uganda	137
Mauritania	138	Tanzania	138	Guatemala	138	Niger	138
Senegal	139	El Salvador	139	Pakistan	139	Gambia	139
Rwanda	140	Mozambique	140	India	140	Burkina Faso	140
Papua New Guinea	141	Guinea	141	Afghanistan	141	Benin	141
Mozambique	142	Afghanistan	142	Uganda	142	Burundi	142
Eritrea	143	Sierra Leone	143	Benin	143	Guinea	143
Togo	144	Senegal	144	Nigeria	144	Angola	144
Burkina Faso	145	Chad	145	Mauritania	145	Zimbabwe	145
Guinea-Bissau	146	Malawi	146	Burkina Faso	146	Mozambique	146
Chad	147	Togo	147	Sierra Leone	147	Togo	147
Somalia	148	Bangladesh	148	Chad	148	Mali	148
Burundi	149	Philippines	149	Nepal	149	Cameroon	149
Sierra Leone	150	Madagascar	150	Senegal	150	Guinea-Bissau	150
Liberia	151	Burundi	151	Niger	151	Somalia	151
Tanzania	152	Chile	152	Guinea	152	Sierra Leone	152
Malawi	153	Niger	153	Madagascar	153	Nigeria	153
Niger	154	Somalia	154	Mali	154	Chad	154

Table S2. Country list corresponding to the index in the horizontal axis of Figure 3.

Asthma		Diabetes		Breast Cancer		AIDS		Influenza and Pneumonia	
**Country**	**Index**	**Country**	**Index**	**Country**	**Index**	**Country**	**Index**	**Country**	**Index**
Sri Lanka	1	Fiji	1	Grenada	1	Malawi	1	Sierra Leone	1
Mali	2	Mauritius	2	Barbados	2	Zimbabwe	2	Nigeria	2
Myanmar	3	Trinidad/Tob.	3	Bahamas	3	South Africa	3	Chad	3
Laos	4	Belize	4	Antigua/Bar.	4	Botswana	4	Angola	4
Fiji	5	Grenada	5	Nigeria	5	Cameroon	5	Niger	5
Sierra Leone	6	Guyana	6	Fiji	6	Mozambique	6	Benin	6
Cambodia	7	Bahrain	7	Pakistan	7	Zambia	7	Somalia	7
Chad	8	Equ. Guinea	8	Syria	8	Namibia	8	Togo	8
Timor-Leste	9	Mexico	9	Eritrea	9	Equ. Guinea	9	Gambia	9
Philippines	10	South Africa	10	Uruguay	10	South Sudan	10	Burkina Faso	10
Madagascar	11	Jamaica	11	Trinidad/Tob.	11	Guinea-Bissau	11	Guinea-Bissau	11
Afghanistan	12	Morocco	12	Afghanistan	12	Congo	12	Guinea	12
Indonesia	13	Tunisia	13	Ethiopia	13	Uganda	13	Ghana	13
Angola	14	Qatar	14	Lebanon	14	Nigeria	14	Cameroon	14
Somalia	15	Guatemala	15	Saint Lucia	15	Togo	15	Equ. Guinea	15
Togo	16	Botswana	16	Serbia	16	Chad	16	Mauritania	16
Sao Tome	17	Saint Lucia	17	Belgium	17	Gabon	17	Burundi	17
Benin	18	Barbados	18	Chad	18	Tanzania	18	Liberia	18
Nepal	19	Venezuela	19	Somalia	19	Kenya	19	South Sudan	19
Niger	20	Brunei	20	Croatia	20	Djibouti	20	Philippines	20
Guinea	21	Angola	21	Iceland	21	Gambia	21	Eritrea	21
Gambia	22	Indonesia	22	Philippines	22	Angola	22	Sao Tome	22
Guinea-Bissau	23	Namibia	23	Denmark	23	Liberia	23	Zimbabwe	23
Equ. Guinea	24	Antigua/Bar.	24	Argentina	24	Sierra Leone	24	Tanzania	24
Cameroon	25	Gabon	25	Cyprus	25	Bahamas	25	Malawi	25
Namibia	26	Suriname	26	Netherlands	26	Guinea	26	Djibouti	26
Eritrea	27	Sierra Leone	27	Jamaica	27	Mali	27	Gabon	27
Burundi	28	Mauritania	28	Iraq	28	Ghana	28	Uganda	28
Nigeria	29	Jordan	29	Israel	29	Belize	29	Senegal	29
South Africa	30	Sri Lanka	30	United Kingdom	30	Haiti	30	Namibia	30
Zimbabwe	31	Haiti	31	Ireland	31	Burundi	31	Rwanda	31
South Sudan	32	Oman	32	Djibouti	32	Rwanda	32	Peru	32
Haiti	33	Ecuador	33	France	33	Sao Tome	33	Zambia	33
Botswana	34	Djibouti	34	Jordan	34	Dominican Rep.	34	Kenya	34
Burkina Faso	35	Bhutan	35	Guyana	35	Jamaica	35	Mozambique	35

Yemen	36	Paraguay	36	Montenegro	36	Guyana	36	Congo	36
Mauritania	37	Ghana	37	Russia	37	Mauritania	37	Ethiopia	37
Ethiopia	38	Cameroon	38	Egypt	38	Somalia	38	Grenada	38
Ghana	39	Nigeria	39	Malta	39	Suriname	39	South Africa	39
Senegal	40	Benin	40	Hungary	40	Burkina Faso	40	Haiti	40
Kazakhstan	41	Zimbabwe	41	Mauritius	41	Niger	41	Madagascar	41
Syria	42	Philippines	42	Germany	42	Ethiopia	42	Botswana	42
India	43	Myanmar	43	Ukraine	43	Benin	43	Mali	43
Bangladesh	44	Nicaragua	44	Slovenia	44	Russia	44	Bolivia	44
Mauritius	45	Iraq	45	South Africa	45	Malaysia	45	Laos	45
Congo	46	Bangladesh	46	Seychelles	46	Thailand	46	Malaysia	46
Uganda	47	Pakistan	47	Slovakia	47	Ukraine	47	Timor-Leste	47
Malawi	48	Gambia	48	Benin	48	Senegal	48	Singapore	48
Sudan	49	Chad	49	Malaysia	49	Myanmar	49	Thailand	49
Liberia	50	Liberia	50	Kenya	50	Trinidad/Tob.	50	Seychelles	50
Djibouti	51	Guinea-Bissau	51	South Sudan	51	Madagascar	51	Guatemala	51
Morocco	52	Laos	52	New Zealand	52	Panama	52	Afghanistan	52
Bhutan	53	Burkina Faso	53	Romania	53	Honduras	53	India	53
Mozambique	54	Panama	54	Luxembourg	54	Cambodia	54	Barbados	54
Viet Nam	55	Egypt	55	Cameroon	55	Paraguay	55	Sudan	55
Rwanda	56	Kuwait	56	Bulgaria	56	Indonesia	56	Belize	56
Tanzania	57	Mali	57	Italy	57	Eritrea	57	Kuwait	57
Gabon	58	Algeria	58	Belize	58	Bhutan	58	Cambodia	58
Zambia	59	Lebanon	59	Latvia	59	Antigua/Bar.	59	Guyana	59
Iran	60	Congo	60	Venezuela	60	Barbados	60	Yemen	60
Pakistan	61	El Salvador	61	Indonesia	61	Belarus	61	Argentina	61
Thailand	62	Kazakhstan	62	Austria	62	Nepal	62	Saudi Arabia	62
Seychelles	63	Nepal	63	Kazakhstan	63	Viet Nam	63	Nepal	63
Kenya	64	Bosnia/Herzeg.	64	Morocco	64	Venezuela	64	Myanmar	64
Tunisia	65	Cyprus	65	Lithuania	65	Guatemala	65	Bhutan	65
Brunei	66	Togo	66	Armenia	66	Sudan	66	Brazil	66
Libya	67	Libya	67	Mali	67	Bolivia	67	El Salvador	67
Bahrain	68	Bahamas	68	Switzerland	68	Ecuador	68	Fiji	68
Malaysia	69	India	69	Algeria	69	El Salvador	69	Pakistan	69
Mongolia	70	Brazil	70	Greece	70	Colombia	70	Oman	70
Uzbekistan	71	Senegal	71	Cuba	71	Mauritius	71	Ecuador	71
Belize	72	Yemen	72	Paraguay	72	Laos	72	Suriname	72
Algeria	73	Tanzania	73	Canada	73	Brazil	73	Morocco	73

Maldives	74	Thailand	74	United States	74	Nicaragua	74	Japan	74
Trinidad/Tob.	75	Armenia	75	Estonia	75	Uruguay	75	Libya	75
Guyana	76	Malawi	76	Moldova	76	Argentina	76	Paraguay	76
Lebanon	77	Mozambique	77	Madagascar	77	Peru	77	Tunisia	77
Dominican Rep.	78	Ethiopia	78	Timor-Leste	78	India	78	Saint Lucia	78
Saudi Arabia	79	South Sudan	79	Albania	79	Latvia	79	Cuba	79
Egypt	80	Sudan	80	Portugal	80	Georgia	80	Antigua/Bar.	80
Antigua/Bar.	81	Uzbekistan	81	Burundi	81	Iran	81	Portugal	81
Guatemala	82	Niger	82	Sudan	82	Estonia	82	Egypt	82
Turkey	83	Eritrea	83	Yemen	83	Portugal	83	Algeria	83
Iraq	84	Dominican Rep.	84	Australia	84	Armenia	84	Russia	84
Jordan	85	Turkey	85	Burkina Faso	85	Mexico	85	Uruguay	85
Nicaragua	86	Guinea	86	Belarus	86	Saint Lucia	86	Indonesia	86
Albania	87	Zambia	87	Sweden	87	Costa Rica	87	Bahamas	87
El Salvador	88	Viet Nam	88	Togo	88	Azerbaijan	88	Kazakhstan	88
Kuwait	89	Cambodia	89	Congo	89	Kyrgyzstan	89	Brunei	89
Suriname	90	Afghanistan	90	Czech Republic	90	Chile	90	Trinidad/Tob.	90
Barbados	91	Burundi	91	Georgia	91	Cuba	91	Nicaragua	91
Uruguay	92	Israel	92	Norway	92	Morocco	92	Uzbekistan	92
Jamaica	93	Somalia	93	Finland	93	Poland	93	Panama	93
Honduras	94	Uganda	94	Brazil	94	Seychelles	94	Romania	94
South Korea	95	Rwanda	95	Costa Rica	95	Greece	95	Belgium	95
Panama	96	Bolivia	96	Uganda	96	Pakistan	96	Venezuela	96
Paraguay	97	Kenya	97	Poland	97	Moldova	97	Sri Lanka	97
Serbia	98	Peru	98	Singapore	98	Grenada	98	Dominican Rep.	98
Azerbaijan	99	Colombia	99	Liberia	99	United States	99	Viet Nam	99
Saint Lucia	100	Seychelles	100	Kuwait	100	Fiji	100	Syria	100
Cyprus	101	Argentina	101	Suriname	101	Yemen	101	United Kingdom	101
Oman	102	Malaysia	102	Panama	102	Kazakhstan	102	Jordan	102
Grenada	103	Timor-Leste	103	Niger	103	Afghanistan	103	Iraq	103
Montenegro	104	Chile	104	Spain	104	Oman	104	Moldova	104
China	105	Azerbaijan	105	Colombia	105	China	105	Mauritius	105
Argentina	106	Serbia	106	Sierra Leone	106	Uzbekistan	106	Bangladesh	106
Cuba	107	Portugal	107	Sao Tome	107	Spain	107	Slovakia	107
Georgia	108	Iran	108	Dominican Rep.	108	Bulgaria	108	Israel	108
Venezuela	109	Croatia	109	Brunei	109	Lebanon	109	Bahrain	109
Bahamas	110	Georgia	110	Turkey	110	Italy	110	Colombia	110
Mexico	111	Saudi Arabia	111	India	111	Romania	111	Chile	111

Ukraine	112	Czech Republic	112	Mauritania	112	Albania	112	South Korea	112
Israel	113	Costa Rica	113	Equ. Guinea	113	Montenegro	113	Malta	113
Brazil	114	South Korea	114	Guinea-Bissau	114	Canada	114	Iran	114
Bosnia/Herzeg.	115	Madagascar	115	Bosnia/Herzeg.	115	Brunei	115	Norway	115
Kyrgyzstan	116	Honduras	116	Ghana	116	Singapore	116	Mexico	116
Australia	117	Austria	117	Haiti	117	Tunisia	117	Azerbaijan	117
Peru	118	Sao Tome	118	Zimbabwe	118	Sri Lanka	118	Maldives	118
Bolivia	119	Uruguay	119	Maldives	119	France	119	Jamaica	119
Chile	120	United States	120	Angola	120	Bangladesh	120	Poland	120
Estonia	121	Montenegro	121	Chile	121	Luxembourg	121	Denmark	121
United Kingdom	122	Italy	122	Bahrain	122	Bahrain	122	Honduras	122
Russia	123	Maldives	123	Uzbekistan	123	Israel	123	Ireland	123
New Zealand	124	Hungary	124	Bangladesh	124	Turkey	124	Mongolia	124
Latvia	125	Cuba	125	Thailand	125	Austria	125	Sweden	125
Belarus	126	Bulgaria	126	Zambia	126	Lithuania	126	Czech Republic	126
France	127	Syria	127	Namibia	127	Germany	127	Slovenia	127
United States	128	Denmark	128	Myanmar	128	Switzerland	128	United States	128
Norway	129	Latvia	129	Tanzania	129	Egypt	129	China	129
Costa Rica	130	Malta	130	Kyrgyzstan	130	Belgium	130	Bulgaria	130
Spain	131	New Zealand	131	Qatar	131	Malta	131	Netherlands	131
Poland	132	Germany	132	Senegal	132	Denmark	132	Turkey	132
Ireland	133	Australia	133	Mexico	133	Algeria	133	France	133
Lithuania	134	Slovakia	134	Tunisia	134	United Kingdom	134	Costa Rica	134
Ecuador	135	Canada	135	Japan	135	Serbia	135	Latvia	135
Colombia	136	Poland	136	Ecuador	136	Timor-Leste	136	Luxembourg	136
Luxembourg	137	China	137	Laos	137	Ireland	137	Iceland	137
Finland	138	Sweden	138	Iran	138	New Zealand	138	Kyrgyzstan	138
Armenia	139	Kyrgyzstan	139	Nicaragua	139	Qatar	139	Lithuania	139
Sweden	140	Spain	140	Sri Lanka	140	Cyprus	140	Albania	140
Romania	141	France	141	Cambodia	141	Australia	141	Qatar	141
Belgium	142	Netherlands	142	Saudi Arabia	142	South Korea	142	Greece	142
Singapore	143	Luxembourg	143	Malawi	143	Syria	143	New Zealand	143
Czech Republic	144	Moldova	144	Azerbaijan	144	Croatia	144	Germany	144
Germany	145	Norway	145	Libya	145	Netherlands	145	Georgia	145
Japan	146	Ireland	146	Oman	146	Norway	146	Spain	146
Malta	147	Slovenia	147	Nepal	147	Mongolia	147	Switzerland	147
Qatar	148	Switzerland	148	Rwanda	148	Sweden	148	Estonia	148
Hungary	149	Belgium	149	El Salvador	149	Philippines	149	Armenia	149

Croatia	150	Romania	150	Honduras	150	Hungary	150	Australia	150
Switzerland	151	Estonia	151	Peru	151	Czech Republic	151	Canada	151
Portugal	152	Lithuania	152	Botswana	152	Finland	152	Ukraine	152
Slovenia	153	Mongolia	153	Mozambique	153	Bosnia/Herzeg.	153	Lebanon	153
Slovakia	154	Greece	154	Guinea	154	Slovenia	154	Serbia	154
Denmark	155	Finland	155	Bolivia	155	Jordan	155	Cyprus	155
Moldova	156	United Kingdom	156	Guatemala	156	Japan	156	Italy	156
Canada	157	Russia	157	Gabon	157	Slovakia	157	Bosnia/Herzeg.	157
Austria	158	Iceland	158	Viet Nam	158	Maldives	158	Croatia	158
Iceland	159	Singapore	159	South Korea	159	Kuwait	159	Montenegro	159
Netherlands	160	Japan	160	China	160	Iraq	160	Hungary	160
Greece	161	Ukraine	161	Gambia	161	Saudi Arabia	161	Belarus	161
Bulgaria	162	Albania	162	Mongolia	162	Libya	162	Austria	162
Italy	163	Belarus	163	Bhutan	163	Iceland	163	Finland	163
**Tuberculosis**		**Stroke**		**Smoke**		**Coronary Heart Disease**		**Hypertension**	
**Country**	**Index**	**Country**	**Index**	**Country**	**Index**	**Country**	**Index**	**Country**	**Index**
Nigeria	1	Indonesia	1	Myanmar	1	Ukraine	1	Seychelles	1
Liberia	2	Sierra Leone	2	Montenegro	2	Kyrgyzstan	2	Uzbekistan	2
Guinea-Bissau	3	Mongolia	3	Ukraine	3	Belarus	3	Guyana	3
Timor-Leste	4	Russia	4	Serbia	4	Uzbekistan	4	Madagascar	4
Sierra Leone	5	Montenegro	5	Azerbaijan	5	Moldova	5	Burundi	5
Mozambique	6	Yemen	6	Bosnia/Herzeg.	6	Yemen	6	Jordan	6
Somalia	7	Ghana	7	Bulgaria	7	Azerbaijan	7	Angola	7
Tanzania	8	Myanmar	8	Laos	8	Russia	8	Bulgaria	8
Angola	9	Afghanistan	9	Philippines	9	Afghanistan	9	Belize	9
Gabon	10	Georgia	10	Zimbabwe	10	Syria	10	Georgia	10
Afghanistan	11	Togo	11	Mongolia	11	Pakistan	11	Haiti	11
Congo	12	Angola	12	Belarus	12	Mongolia	12	Iran	12
Laos	13	Benin	13	Russia	13	Lithuania	13	Mozambique	13
Madagascar	14	Guyana	14	Cambodia	14	Georgia	14	Uganda	14
Cambodia	15	Haiti	15	Pakistan	15	Sudan	15	Eritrea	15
Ghana	16	Mali	16	Hungary	16	Egypt	16	Rwanda	16
Zambia	17	Kyrgyzstan	17	Nepal	17	Iraq	17	South Africa	17
Guinea	18	Gambia	18	Egypt	18	Lebanon	18	Malawi	18
Bangladesh	19	Sudan	19	Fiji	19	Armenia	19	Equ. Guinea	19
South Africa	20	Cameroon	20	China	20	Romania	20	Bahamas	20
Namibia	21	Philippines	21	Kazakhstan	21	Bulgaria	21	Grenada	21
Djibouti	22	Chad	22	Armenia	22	Guyana	22	Botswana	22

Myanmar	23	Bulgaria	23	Indonesia	23	Latvia	23	Kuwait	23
Cameroon	24	China	24	Georgia	24	Kuwait	24	Burkina Faso	24
Chad	25	Bangladesh	25	Lebanon	25	Libya	25	Antigua/Bar.	25
Burundi	26	Guinea-Bissau	26	Moldova	26	Philippines	26	South Sudan	26
South Sudan	27	Madagascar	27	Romania	27	Sierra Leone	27	Namibia	27
Indonesia	28	Laos	28	Timor-Leste	28	Kazakhstan	28	Myanmar	28
Botswana	29	Eritrea	29	Poland	29	Hungary	29	Tanzania	29
Senegal	30	Moldova	30	Latvia	30	Estonia	30	Gabon	30
Ethiopia	31	Suriname	31	Croatia	31	Saudi Arabia	31	Ecuador	31
Gambia	32	Nigeria	32	Brunei	32	Iran	32	Ethiopia	32
India	33	Senegal	33	Yemen	33	Burkina Faso	33	Somalia	33
Philippines	34	Cambodia	34	Lithuania	34	Laos	34	Fiji	34
Niger	35	Ukraine	35	Greece	35	Haiti	35	Djibouti	35
Kenya	36	Belarus	36	Malaysia	36	Fiji	36	Congo	36
Mauritania	37	Guinea	37	Viet Nam	37	Nepal	37	Saint Lucia	37
Pakistan	38	Viet Nam	38	Uzbekistan	38	Slovakia	38	Philippines	38
Sudan	39	Niger	39	Albania	39	Ghana	39	Jamaica	39
Uganda	40	Sao Tome	40	Czech Republic	40	Trinidad/Tob.	40	Moldova	40
Mongolia	41	Equ. Guinea	41	Cuba	41	Czech Republic	41	Kenya	41
Malawi	42	Mauritania	42	Denmark	42	Grenada	42	Sierra Leone	42
Eritrea	43	Mozambique	43	Argentina	43	Bhutan	43	Zambia	43
Nepal	44	Romania	44	Tunisia	44	Benin	44	Romania	44
Haiti	45	Liberia	45	India	45	Croatia	45	Ghana	45
Zimbabwe	46	Syria	46	Mozambique	46	Poland	46	Trinidad/Tob.	46
Mali	47	Albania	47	Slovakia	47	Montenegro	47	Mauritius	47
Burkina Faso	48	Latvia	48	Afghanistan	48	India	48	Estonia	48
Bhutan	49	Azerbaijan	49	Namibia	49	Togo	49	Suriname	49
Benin	50	Gabon	50	Uruguay	50	Cambodia	50	China	50
Viet Nam	51	Bosnia/Herzeg.	51	Turkey	51	Mali	51	Laos	51
Guyana	52	Timor-Leste	52	Kyrgyzstan	52	Gambia	52	Yemen	52
Libya	53	Congo	53	Netherlands	53	Indonesia	53	Afghanistan	53
Kyrgyzstan	54	Burundi	54	Angola	54	Malaysia	54	Hungary	54
Equ. Guinea	55	Pakistan	55	Dominican Rep.	55	Bahrain	55	Sudan	55
Kazakhstan	56	Tunisia	56	Paraguay	56	Jordan	56	India	56
Togo	57	Botswana	57	Seychelles	57	Dominican Rep.	57	Venezuela	57
Ukraine	58	Egypt	58	Belgium	58	Guinea-Bissau	58	Cambodia	58
Morocco	59	Burkina Faso	59	Rwanda	59	Angola	59	Togo	59
Russia	60	Iraq	60	Suriname	60	Oman	60	Mali	60

Uzbekistan	61	Grenada	61	Estonia	61	Sri Lanka	61	Serbia	61
Thailand	62	South Africa	62	Syria	62	Equ. Guinea	62	Turkey	62
Malaysia	63	Djibouti	63	Somalia	63	Venezuela	63	Nigeria	63
Algeria	64	Uganda	64	Bangladesh	64	Mauritania	64	Nepal	64
Brunei	65	Namibia	65	Cyprus	65	Albania	65	Egypt	65
Sao Tome	66	Somalia	66	Zambia	66	Chad	66	Cameroon	66
Fiji	67	Kazakhstan	67	Ireland	67	Morocco	67	Barbados	67
Maldives	68	Ethiopia	68	United States	68	Cameroon	68	Dominican Rep.	68
Yemen	69	Saudi Arabia	69	Jamaica	69	Belize	69	Pakistan	69
Rwanda	70	Libya	70	Brazil	70	Bosnia/Herzeg.	70	Armenia	70
Belarus	71	South Sudan	71	Botswana	71	Timor-Leste	71	Indonesia	71
Peru	72	Lithuania	72	Germany	72	Serbia	72	Gambia	72
Panama	73	Uzbekistan	73	Gambia	73	Suriname	73	Benin	73
Belize	74	Iran	74	Sierra Leone	74	Nigeria	74	Chad	74
Moldova	75	Kuwait	75	Canada	75	Niger	75	Algeria	75
Iraq	76	Armenia	76	Honduras	76	Namibia	76	Nicaragua	76
Dominican Rep.	77	Rwanda	77	Mauritius	77	Guinea	77	Paraguay	77
Sri Lanka	78	Serbia	78	South Africa	78	Mauritius	78	Bhutan	78
Saudi Arabia	79	Saint Lucia	79	Tanzania	79	South Africa	79	Guinea	79
Paraguay	80	Nepal	80	United Kingdom	80	Seychelles	80	Timor-Leste	80
Lithuania	81	Belize	81	Slovenia	81	Greece	81	Iraq	81
Ecuador	82	Jordan	82	Malta	82	Senegal	82	Niger	82
South Korea	83	Morocco	83	South Sudan	83	Maldives	83	Guinea-Bissau	83
Romania	84	Jamaica	84	Djibouti	84	Liberia	84	Honduras	84
Bolivia	85	Paraguay	85	Luxembourg	85	Algeria	85	Chile	85
Nicaragua	86	Tanzania	86	Burundi	86	Bangladesh	86	Latvia	86
Georgia	87	Croatia	87	Congo	87	Turkey	87	Senegal	87
Guatemala	88	Trinidad/Tob.	88	Guyana	88	Honduras	88	Morocco	88
Colombia	89	Algeria	89	Jordan	89	Botswana	89	Liberia	89
Venezuela	90	Sri Lanka	90	Spain	90	Tunisia	90	Colombia	90
Suriname	91	Fiji	91	Maldives	91	Argentina	91	Austria	91
Latvia	92	Malawi	92	Austria	92	Ethiopia	92	Croatia	92
Brazil	93	Malaysia	93	Haiti	93	Malta	93	Mauritania	93
Armenia	94	India	94	Libya	94	El Salvador	94	Argentina	94
Mexico	95	Dominican Rep.	95	Gabon	95	Congo	95	Sri Lanka	95
Iran	96	Barbados	96	Algeria	96	Qatar	96	Viet Nam	96
China	97	Kenya	97	Norway	97	Paraguay	97	Libya	97
Tunisia	98	Bhutan	98	Iran	98	Cuba	98	Brazil	98

Chile	99	Greece	99	Venezuela	99	Nicaragua	99	Cuba	99
Honduras	100	Zambia	100	Morocco	100	China	100	Costa Rica	100
El Salvador	101	Zimbabwe	101	Thailand	101	Gabon	101	Italy	101
Trinidad/Tob.	102	Bolivia	102	Iceland	102	Colombia	102	Panama	102
Bosnia/Herzeg.	103	Poland	103	Sweden	103	Sao Tome	103	Zimbabwe	103
Saint Lucia	104	Thailand	104	South Korea	104	Austria	104	Kyrgyzstan	104
Estonia	105	Turkey	105	France	105	Eritrea	105	Bangladesh	105
Argentina	106	Hungary	106	Grenada	106	Bolivia	106	Cyprus	106
Uruguay	107	Lebanon	107	Trinidad/Tob.	107	Mexico	107	Slovakia	107
Mauritius	108	Seychelles	108	Italy	108	Cyprus	108	Switzerland	108
Bahrain	109	Venezuela	109	Malawi	109	Madagascar	109	Germany	109
Qatar	110	Slovakia	110	Sri Lanka	110	Antigua/Bar.	110	Mongolia	110
Portugal	111	Mauritius	111	Saint Lucia	111	United States	111	El Salvador	111
Costa Rica	112	Brazil	112	Madagascar	112	Djibouti	112	Slovenia	112
Lebanon	113	Honduras	113	Bhutan	113	Bahamas	113	Mexico	113
Oman	114	Nicaragua	114	Iraq	114	Finland	114	Singapore	114
Turkey	115	Antigua/Bar.	115	New Zealand	115	Slovenia	115	Czech Republic	115
Bulgaria	116	Portugal	116	Switzerland	116	Tanzania	116	Lebanon	116
Poland	117	Cuba	117	Israel	117	Malawi	117	Tunisia	117
Japan	118	Brunei	118	Japan	118	Zambia	118	Guatemala	118
Serbia	119	Maldives	119	Belize	119	Guatemala	119	United States	119
Singapore	120	Bahamas	120	Oman	120	Germany	120	Uruguay	120
Grenada	121	Uruguay	121	Portugal	121	Iceland	121	Azerbaijan	121
Iceland	122	Oman	122	Australia	122	Brunei	122	Greece	122
Bahamas	123	Argentina	123	Liberia	123	Brazil	123	Kazakhstan	123
Jordan	124	Czech Republic	124	Kenya	124	Burundi	124	Finland	124
Hungary	125	Panama	125	Bolivia	125	Myanmar	125	Sweden	125
Antigua/Bar.	126	Slovenia	126	Eritrea	126	Mozambique	126	Lithuania	126
Kuwait	127	Guatemala	127	Mexico	127	Zimbabwe	127	Poland	127
Slovenia	128	Chile	128	Cameroon	128	Rwanda	128	Spain	128
Greece	129	Bahrain	129	Bahrain	129	Sweden	129	Russia	129
Croatia	130	Colombia	130	Benin	130	Barbados	130	Belarus	130
France	131	Peru	131	Finland	131	Peru	131	Ukraine	131
Austria	132	Ecuador	132	Costa Rica	132	South Sudan	132	Bahrain	132
Azerbaijan	133	Italy	133	Chile	133	Saint Lucia	133	Syria	133
Jamaica	134	Estonia	134	Guinea-Bissau	134	Panama	134	Portugal	134
Italy	135	South Korea	135	Togo	135	Costa Rica	135	Israel	135
Spain	136	Finland	136	Uganda	136	New Zealand	136	Sao Tome	136

Slovakia	137	Japan	137	Bahamas	137	Ireland	137	Bosnia/Herzeg.	137
Finland	138	Mexico	138	Guinea	138	Thailand	138	South Korea	138
Belgium	139	Cyprus	139	Kuwait	139	Uruguay	139	Maldives	139
Egypt	140	New Zealand	140	Ghana	140	Viet Nam	140	Oman	140
Czech Republic	141	Malta	141	Sao Tome	141	Somalia	141	Denmark	141
Albania	142	Qatar	142	Equ. Guinea	142	Uganda	142	Saudi Arabia	142
United Kingdom	143	El Salvador	143	El Salvador	143	Italy	143	Bolivia	143
Montenegro	144	Costa Rica	144	Panama	144	Jamaica	144	Iceland	144
Cuba	145	Norway	145	Antigua/Bar.	145	Norway	145	Brunei	145
Ireland	146	Belgium	146	Qatar	146	Singapore	146	Peru	146
Norway	147	Sweden	147	Guatemala	147	Switzerland	147	France	147
Cyprus	148	Netherlands	148	Burkina Faso	148	Belgium	148	Thailand	148
Denmark	149	United Kingdom	149	Colombia	149	Ecuador	149	Luxembourg	149
Germany	150	Denmark	150	Singapore	150	Luxembourg	150	Australia	150
New Zealand	151	Iceland	151	Saudi Arabia	151	United Kingdom	151	Norway	151
Israel	152	Ireland	152	Mali	152	Canada	152	Albania	152
Canada	153	Luxembourg	153	Barbados	153	Australia	153	Malaysia	153
Syria	154	Germany	154	Senegal	154	Netherlands	154	New Zealand	154
Switzerland	155	Spain	155	Nicaragua	155	Spain	155	Ireland	155
Barbados	156	Austria	156	Ecuador	156	Chile	156	Netherlands	156
Sweden	157	Australia	157	Chad	157	Portugal	157	United Kingdom	157
United States	158	United States	158	Mauritania	158	Israel	158	Montenegro	158
Netherlands	159	Singapore	159	Ethiopia	159	Denmark	159	Belgium	159
Australia	160	France	160	Niger	160	Kenya	160	Canada	160
Malta	161	Israel	161	Peru	161	France	161	Qatar	161
Luxembourg	162	Switzerland	162	Nigeria	162	Japan	162	Malta	162
Seychelles	163	Canada	163	Sudan	163	South Korea	163	Japan	163

Table S3. Country list corresponding to the index in the horizontal axis of Figure 4.

Prevalence of Asthma		Prevalence of Diabetes		Prevalence of Breast Cancer		Prevalence of AIDS	
**Country**	**Index**	**Country**	**Index**	**Country**	**Index**	**Country**	**Index**
Papua New Guinea	1	New Caledonia	1	China	1	Botswana	1
Australia	2	French Polynesia	2	United States	2	South Africa	2
Fiji	3	Mauritius	3	India	3	Zimbabwe	3
United Kingdom	4	Saudi Arabia	4	Japan	4	Mozambique	4
Bermuda	5	Papua New Guinea	5	Germany	5	Namibia	5
Iceland	6	Egypt	6	Russia	6	Zambia	6
Portugal	7	United Arab Emirates	7	United Kingdom	7	Malawi	7
Sweden	8	Belize	8	Italy	8	Equatorial Guinea	8
Trinidad and Tobago	9	Malaysia	9	France	9	Uganda	9
Norway	10	Bahrain	10	Brazil	10	Kenya	10
New Zealand	11	Qatar	11	Indonesia	11	Tanzania	11
Ireland	12	Kuwait	12	Pakistan	12	Gabon	12
Suriname	13	Sudan	13	Spain	13	Cameroon	13
Cuba	14	Fiji	14	Canada	14	Guinea-Bissau	14
Bahamas	15	British Virgin Islands	15	Mexico	15	Congo	15
Belize	16	Barbados	16	Nigeria	16	Nigeria	16
Grenada	17	Cayman Islands	17	Philippines	17	Rwanda	17
Saint Lucia	18	Antigua and Barbuda	18	South Korea	18	South Sudan	18
Jamaica	19	Bahamas	19	Poland	19	Togo	19
Antigua and Barbuda	20	Mexico	20	Ukraine	20	Angola	20
Lebanon	21	Bermuda	21	Thailand	21	Bahamas	21
Dominica	22	Saint Kitts and Nevis	22	Turkey	22	Belize	22
United Arab Emirates	23	Brunei	23	Netherlands	23	Haiti	23
Guyana	24	Lebanon	24	Australia	24	Jamaica	24
Luxembourg	25	Oman	25	Iran	25	Ghana	25
Netherlands	26	Suriname	26	Argentina	26	Guyana	26
Haiti	27	Turkey	27	Vietnam	27	Barbados	27
Barbados	28	Jordan	28	Bangladesh	28	Gambia	28
Jordan	29	Aruba	29	Taiwan	29	Guinea	29
Egypt	30	Curacao	30	Myanmar	30	Liberia	30
Andorra	31	Dominica	31	Colombia	31	Sierra Leone	31
France	32	Guyana	32	Egypt	32	Chad	32
Kuwait	33	Saint Lucia	33	Belgium	33	Djibouti	33
Greenland	34	Nicaragua	34	Romania	34	Suriname	34

Philippines	35	Jamaica	35	Sweden	35	Mali	35
Bolivia	36	Singapore	36	Greece	36	Russia	36
Paraguay	37	Trinidad and Tobago	37	Morocco	37	Burundi	37
Afghanistan	38	United States	38	Malaysia	38	Thailand	38
Finland	39	Grenada	39	Venezuela	39	Trinidad and Tobago	39
Switzerland	40	Sri Lanka	40	Portugal	40	Benin	40
Algeria	41	Palestine	41	South Africa	41	Panama	41
Malta	42	Seychelles	42	Algeria	42	Dominican Republic	42
Congo	43	Libya	43	Switzerland	43	Ethiopia	43
Palestine	44	South Sudan	44	Austria	44	Papua New Guinea	44
Mauritius	45	India	45	Czech Republic	45	Ukraine	45
Costa Rica	46	Guatemala	46	Hungary	46	Burkina Faso	46
Canada	47	Albania	47	Saudi Arabia	47	Estonia	47
Dominican Republic	48	Bosnia and Herzegovina	48	Ethiopia	48	Myanmar	48
Rwanda	49	Montenegro	49	Serbia	49	Brazil	49
Uruguay	50	Serbia	50	Chile	50	Chile	50
Denmark	51	Portugal	51	Cuba	51	El Salvador	51
Iraq	52	Bhutan	52	Lebanon	52	Eritrea	52
Gabon	53	China	53	Uzbekistan	53	Moldova	53
Sudan	54	Romania	54	Finland	54	Portugal	54
Cyprus	55	Afghanistan	55	Denmark	55	Uruguay	55
Israel	56	Iran	56	Bulgaria	56	Cambodia	56
Tanzania	57	Cyprus	57	Israel	57	Colombia	57
Equatorial Guinea	58	Maldives	58	Peru	58	France	58
Libya	59	El Salvador	59	Sri Lanka	59	Paraguay	59
United States	60	Iraq	60	Kazakhstan	60	Argentina	60
Thailand	61	Malta	61	Iraq	61	Belarus	61
Bahrain	62	Costa Rica	62	Belarus	62	Costa Rica	62
Panama	63	Tunisia	63	Ireland	63	Cuba	63
Austria	64	Chile	64	Norway	64	Georgia	64
Greece	65	Bangladesh	65	New Zealand	65	Guatemala	65
Sri Lanka	66	Pakistan	66	Tanzania	66	Indonesia	66
Indonesia	67	Hong Kong	67	Tunisia	67	Malaysia	67
El Salvador	68	Panama	68	Kenya	68	Senegal	68
Spain	69	Germany	69	Slovakia	69	Spain	69
Syria	70	Cuba	70	Croatia	70	Bolivia	70
Peru	71	Paraguay	71	Ghana	71	Ecuador	71
Angola	72	Dominican Republic	72	Syria	72	Honduras	72

Turkey	73	Brazil	73	Singapore	73	Laos	73
Seychelles	74	New Zealand	74	Nepal	74	Luxembourg	74
Laos	75	Andorra	75	Ecuador	75	Madagascar	75
Honduras	76	Equatorial Guinea	76	Azerbaijan	76	Mauritania	76
Chile	77	Liechtenstein	77	Sudan	77	Mexico	77
Ecuador	78	Uzbekistan	78	Dominican Republic	78	Niger	78
Maldives	79	Hungary	79	Uganda	79	Peru	79
Taiwan	80	Ethiopia	80	Cambodia	80	Uzbekistan	80
Brazil	81	Colombia	81	Uruguay	81	Vietnam	81
Myanmar	82	Canada	82	Jordan	82	Armenia	82
Tunisia	83	Slovakia	83	Georgia	83	Germany	83
Argentina	84	Nepal	84	Afghanistan	84	Greece	84
Oman	85	Slovenia	85	Mozambique	85	India	85
Madagascar	86	Honduras	86	Yemen	86	Ireland	86
Poland	87	Cameroon	87	Libya	87	Italy	87
Cambodia	88	Congo	88	Cameroon	88	Kazakhstan	88
Brunei	89	Gabon	89	Lithuania	89	Kyrgyzstan	89
Yemen	90	Spain	90	Costa Rica	90	Lithuania	90
Germany	91	Morocco	91	Burkina Faso	91	Nepal	91
Belgium	92	Armenia	92	Angola	92	Netherlands	92
Uganda	93	Azerbaijan	93	Bosnia and Herzegovina	93	Nicaragua	93
Malaysia	94	Georgia	94	Madagascar	94	Singapore	94
Japan	95	Kazakhstan	95	Armenia	95	Sudan	95
South Korea	96	Kyrgyzstan	96	Bolivia	96	Albania	96
Qatar	97	Ukraine	97	Moldova	97	Algeria	97
Singapore	98	Philippines	98	Slovenia	98	Australia	98
Slovenia	99	Thailand	99	Haiti	99	Austria	99
Romania	100	Uruguay	100	Guatemala	100	Azerbaijan	100
South Sudan	101	Bolivia	101	United Arab Emirates	101	Bahrain	101
Iran	102	Czech Republic	102	Paraguay	102	Bangladesh	102
Morocco	103	South Korea	103	El Salvador	103	Bulgaria	103
Mauritania	104	Israel	104	Latvia	104	Cyprus	104
Venezuela	105	Algeria	105	Mali	105	Czech Republic	105
Bosnia and Herzegovina	106	Haiti	106	Jamaica	106	Denmark	106
Mozambique	107	Venezuela	107	Papua New Guinea	107	Egypt	107
Djibouti	108	Denmark	108	Zimbabwe	108	Hungary	108
Malawi	109	Austria	109	Zambia	109	Iran	109
Belarus	110	Indonesia	110	Cyprus	110	Japan	110

Uzbekistan	111	Russia	111	Panama	111	Kuwait	111
Colombia	112	Togo	112	Senegal	112	Lebanon	112
Nicaragua	113	Chad	113	Rwanda	113	Mongolia	113
Vietnam	114	Burundi	114	Honduras	114	Montenegro	114
Kyrgyzstan	115	Djibouti	115	Albania	115	Morocco	115
Burundi	116	Eritrea	116	Estonia	116	New Zealand	116
Guinea	117	Somalia	117	Nicaragua	117	Norway	117
Eritrea	118	Vietnam	118	Palestine	118	Pakistan	118
Latvia	119	Peru	119	Somalia	119	Philippines	119
Croatia	120	Poland	120	Laos	120	Qatar	120
Pakistan	121	Bulgaria	121	Kyrgyzstan	121	Romania	121
Ukraine	122	Finland	122	Malawi	122	Serbia	122
Somalia	123	Tanzania	123	Kuwait	123	Slovakia	123
Nepal	124	Japan	124	Guinea	124	Slovenia	124
Montenegro	125	Moldova	125	Niger	125	Somalia	125
Zambia	126	San Marino	126	Mauritius	126	Sri Lanka	126
Mongolia	127	Croatia	127	Benin	127	Tunisia	127
Ethiopia	128	Switzerland	128	Congo	128		
Burkina Faso	129	Ecuador	129	Luxembourg	129		
Togo	130	South Africa	130	Trinidad and Tobago	130		
Botswana	131	Argentina	131	Togo	131		
Italy	132	Monaco	132	Chad	132		
Sao Tome and Principe	133	Yemen	133	Eritrea	133		
Saudi Arabia	134	Iceland	134	Burundi	134		
Mexico	135	Norway	135	Sierra Leone	135		
Guatemala	136	Netherlands	136	South Sudan	136		
Armenia	137	Belarus	137	Malta	137		
Azerbaijan	138	Australia	138	Oman	138		
Bulgaria	139	Ghana	139	Qatar	139		
Namibia	140	Latvia	140	Montenegro	140		
Moldova	141	Mongolia	141	Namibia	141		
Kenya	142	Botswana	142	Mauritania	142		
Lithuania	143	Sweden	143	Botswana	143		
Benin	144	Italy	144	Mongolia	144		
Guinea-Bissau	145	France	145	Bahrain	145		
Cameroon	146	Myanmar	146	Liberia	146		
Georgia	147	Greece	147	Fiji	147		
Hungary	148	Luxembourg	148	Iceland	148		

Czech Republic	149	Belgium	149	Bahamas	149		
Sierra Leone	150	Channel Islands	150	Barbados	150		
Liberia	151	Rwanda	151	Gabon	151		
Gambia	152	United Kingdom	152	Brunei	152		
Bhutan	153	Estonia	153	Guyana	153		
Niger	154	Cambodia	154	Suriname	154		
Kazakhstan	155	Laos	155	Equatorial Guinea	155		
Serbia	156	Angola	156	Guinea-Bissau	156		
Slovakia	157	Madagascar	157	Djibouti	157		
Mali	158	Malawi	158	Gambia	158		
Nigeria	159	Namibia	159	Bhutan	159		
Russia	160	Zambia	160	Andorra	160		
Zimbabwe	161	Lithuania	161	Saint Lucia	161		
Chad	162	Mozambique	162	Maldives	162		
Senegal	163	Ireland	163	Bermuda	163		
Albania	164	Kenya	164	Grenada	164		
Bangladesh	165	Uganda	165	Antigua and Barbuda	165		
Ghana	166	Burkina Faso	166	Belize	166		
China	167	Guinea	167	Seychelles	167		
Estonia	168	Guinea-Bissau	168	Dominica	168		
India	169	Liberia	169	Sao Tome and Principe	169		
South Africa	170	Mali	170	Greenland	170		
		Mauritania	171				
		Niger	172				
		Nigeria	173				
		Sao Tome and Principe	174				
		Senegal	175				
		Sierra Leone	176				
		Greenland	177				
		Gambia	178				
		Zimbabwe	179				
		Benin	180				

**Prevalence of Liver Cancer**		**Prevalence TB**		**Prevalence of Stomach Cancer**		**Prevalence of Smoker Aged 10 and Older**	
**Country**	**Index**	**Country**	**Index**	**Country**	**Index**	**Country**	**Index**
China	1	India	1	China	1	Montenegro	1
Japan	2	China	2	Japan	2	Bosnia and Herzegovina	2
South Korea	3	Indonesia	3	India	3	Bulgaria	3
United States	4	Nigeria	4	United States	4	Greece	4

Taiwan	5	Brazil	5	Russia	5	Serbia	5
Thailand	6	Vietnam	6	South Korea	6	Croatia	6
Italy	7	Russia	7	Germany	7	Latvia	7
Germany	8	Pakistan	8	Italy	8	Ukraine	8
Vietnam	9	Mexico	9	Brazil	9	Laos	9
Egypt	10	Philippines	10	United Kingdom	10	Russia	10
Spain	11	Bangladesh	11	Spain	11	Indonesia	11
France	12	Egypt	12	Ukraine	12	Papua New Guinea	12
United Kingdom	13	Thailand	13	Indonesia	13	Lebanon	13
Indonesia	14	South Africa	14	Mexico	14	Hungary	14
Mongolia	15	United States	15	France	15	Belarus	15
Philippines	16	Sudan	16	Colombia	16	Chile	16
Mali	17	Ethiopia	17	Canada	17	Austria	17
Mozambique	18	Myanmar	18	Taiwan	18	Spain	18
Guinea	19	Turkey	19	Iran	19	Cyprus	19
Singapore	20	Ukraine	20	Turkey	20	Lithuania	20
Austria	21	Iran	21	Vietnam	21	France	21
Canada	22	Kenya	22	Bangladesh	22	Poland	22
Australia	23	Uganda	23	Australia	23	Czech Republic	23
Switzerland	24	Japan	24	Poland	24	Romania	24
Niger	25	Tanzania	25	Netherlands	25	Mongolia	25
Ghana	26	Colombia	26	Pakistan	26	Jordan	26
Myanmar	27	Morocco	27	Argentina	27	Georgia	27
Finland	28	Iraq	28	Thailand	28	Japan	28
Romania	29	Saudi Arabia	29	Venezuela	29	Armenia	29
Cameroon	30	Poland	30	Portugal	30	Slovenia	30
Mexico	31	Malaysia	31	Chile	31	Maldives	31
Guatemala	32	Germany	32	Peru	32	Andorra	32
Senegal	33	Afghanistan	33	Myanmar	33	Estonia	33
Burkina Faso	34	Taiwan	34	Afghanistan	34	Uruguay	34
Gambia	35	Venezuela	35	Belarus	35	Philippines	35
Pakistan	36	Yemen	36	Romania	36	Germany	36
Zimbabwe	37	Nepal	37	Greece	37	China	37
Malaysia	38	Italy	38	Austria	38	Tunisia	38
Croatia	39	Mozambique	39	Nigeria	39	Bangladesh	39
Denmark	40	Madagascar	40	Philippines	40	Turkey	40
Chad	41	Cameroon	41	Costa Rica	41	Luxembourg	41
Sweden	42	France	42	Uzbekistan	42	Italy	42

New Zealand	43	United Kingdom	43	Sudan	43	United Kingdom	43
Netherlands	44	South Korea	44	Ecuador	44	Ireland	44
Benin	45	Peru	45	Belgium	45	Slovakia	45
Belgium	46	Angola	46	Yemen	46	Kazakhstan	46
Moldova	47	Ghana	47	Kazakhstan	47	Switzerland	47
Greece	48	Algeria	48	Ethiopia	48	Kyrgyzstan	48
Portugal	49	Syria	49	Czech Republic	49	Palestine	49
Chile	50	Burkina Faso	50	Slovakia	50	South Korea	50
Russia	51	Romania	51	Guatemala	51	Belgium	51
Czech Republic	52	Uzbekistan	52	Bolivia	52	Syria	52
Slovakia	53	Zambia	53	Kenya	53	Cambodia	53
Saudi Arabia	54	Cambodia	54	Egypt	54	Moldova	54
Hungary	55	Niger	55	South Africa	55	Azerbaijan	55
Sierra Leone	56	Tunisia	56	Switzerland	56	Nepal	56
Togo	57	Sri Lanka	57	Azerbaijan	57	Malaysia	57
Azerbaijan	58	Mali	58	Malaysia	58	Portugal	58
India	59	Kazakhstan	59	Nepal	59	Argentina	59
Papua New Guinea	60	Senegal	60	Hungary	60	Egypt	60
Liberia	61	Chad	61	Sweden	61	Malta	61
Bulgaria	62	Guatemala	62	Algeria	62	Denmark	62
Laos	63	Argentina	63	Serbia	63	Thailand	63
Armenia	64	Malawi	64	Tanzania	64	Vietnam	64
Uganda	65	South Sudan	65	Croatia	65	Albania	65
Kazakhstan	66	Belarus	66	Singapore	66	Myanmar	66
Norway	67	Rwanda	67	Bulgaria	67	Israel	67
Guinea-Bissau	68	Ecuador	68	Finland	68	Mauritius	68
Angola	69	Burundi	69	Cuba	69	Finland	69
Slovenia	70	Guinea	70	Mali	70	New Zealand	70
Cambodia	71	Zimbabwe	71	Cameroon	71	Netherlands	71
Mauritania	72	Cuba	72	Lithuania	72	Kuwait	72
Albania	73	Spain	73	Morocco	73	Djibouti	73
Somalia	74	Benin	74	Ireland	74	Yemen	74
Ireland	75	Czech Republic	75	Sri Lanka	75	Suriname	75
Georgia	76	Hungary	76	Ghana	76	Cuba	76
South Africa	77	Canada	77	Cambodia	77	Iraq	77
Kyrgyzstan	78	United Arab Emirates	78	Burkina Faso	78	Libya	78
Luxembourg	79	Haiti	79	Denmark	79	Norway	79
Kenya	80	Dominican Republic	80	El Salvador	80	Seychelles	80

Zambia	81	Papua New Guinea	81	Panama	81	Sierra Leone	81
Brunei	82	Serbia	82	Norway	82	Trinidad and Tobago	82
Gabon	83	Australia	83	Haiti	83	Iceland	83
Bosnia and Herzegovina	84	Bolivia	84	New Zealand	84	Australia	84
Fiji	85	Libya	85	Saudi Arabia	85	Canada	85
Iceland	86	Azerbaijan	86	Latvia	86	Fiji	86
Nicaragua	87	Sierra Leone	87	Israel	87	South Africa	87
Lithuania	88	Bulgaria	88	Georgia	88	Pakistan	88
Brazil	89	Honduras	89	Senegal	89	Qatar	89
Cyprus	90	Togo	90	Niger	90	Brunei	90
Equatorial Guinea	91	Netherlands	91	Papua New Guinea	91	Taiwan	91
Qatar	92	Oman	92	Mongolia	92	United States	92
Congo	93	Chile	93	Guinea	93	Botswana	93
Eritrea	94	Somalia	94	Estonia	94	Zambia	94
Ukraine	95	Laos	95	Madagascar	95	India	95
Ethiopia	96	Paraguay	96	Angola	96	Zimbabwe	96
Montenegro	97	El Salvador	97	Uruguay	97	Saudi Arabia	97
Suriname	98	Nicaragua	98	Iraq	98	Namibia	98
Greenland	99	Kuwait	99	Zimbabwe	99	United Arab Emirates	99
Andorra	100	Palestine	100	Uganda	100	Mexico	100
Botswana	101	Portugal	101	Slovenia	101	Guyana	101
Colombia	102	Belgium	102	Mozambique	102	Malawi	102
Iran	103	Sweden	103	Dominican Republic	103	Iran	103
Turkey	104	Costa Rica	104	Chad	104	Madagascar	104
Bangladesh	105	Greece	105	Kyrgyzstan	105	Venezuela	105
Poland	106	Slovakia	106	Lebanon	106	Mozambique	106
Argentina	107	Liberia	107	Tunisia	107	Algeria	107
Venezuela	108	Eritrea	108	Moldova	108	Morocco	108
Peru	109	Congo	109	Somalia	109	Sudan	109
Afghanistan	110	Kyrgyzstan	110	Bosnia and Herzegovina	110	Afghanistan	110
Belarus	111	Austria	111	Armenia	111	Paraguay	111
Nigeria	112	Moldova	112	Syria	112	Sweden	112
Costa Rica	113	Lebanon	113	Benin	113	Colombia	113
Uzbekistan	114	Croatia	114	Nicaragua	114	Nicaragua	114
Sudan	115	Panama	115	Zambia	115	Jamaica	115
Ecuador	116	Mauritania	116	Albania	116	Gambia	116
Yemen	117	Georgia	117	Paraguay	117	Sri Lanka	117
Bolivia	118	Bosnia and Herzegovina	118	Honduras	118	Gabon	118

Nepal	119	Qatar	119	Sierra Leone	119	Mauritania	119
Algeria	120	Switzerland	120	Togo	120	Saint Lucia	120
Serbia	121	Lithuania	121	Laos	121	Bahrain	121
Tanzania	122	Namibia	122	Rwanda	122	Honduras	122
Cuba	123	Denmark	123	Libya	123	Tanzania	123
Morocco	124	Botswana	124	Burundi	124	Uganda	124
Sri Lanka	125	Jamaica	125	South Sudan	125	Burkina Faso	125
El Salvador	126	Gambia	126	United Arab Emirates	126	Angola	126
Panama	127	Armenia	127	Jamaica	127	Bolivia	127
Haiti	128	Israel	128	Jordan	128	Dominican Republic	128
Latvia	129	Finland	129	Malawi	129	Uzbekistan	129
Israel	130	Mongolia	130	Eritrea	130	Guinea	130
Estonia	131	Mauritius	131	Cyprus	131	Somalia	131
Madagascar	132	Latvia	132	Liberia	132	Kenya	132
Uruguay	133	Bahrain	133	Congo	133	Congo	133
Iraq	134	Guinea-Bissau	134	Mauritania	134	Oman	134
Dominican Republic	135	Albania	135	Oman	135	Grenada	135
Lebanon	136	Jordan	136	Luxembourg	136	Brazil	136
Tunisia	137	Gabon	137	Palestine	137	South Sudan	137
Syria	138	Norway	138	Mauritius	138	Singapore	138
Paraguay	139	Ireland	139	Guinea-Bissau	139	Costa Rica	139
Honduras	140	Slovenia	140	Malta	140	Mali	140
Rwanda	141	Trinidad and Tobago	141	Iceland	141	Guinea-Bissau	141
Libya	142	New Zealand	142	Trinidad and Tobago	142	El Salvador	142
Burundi	143	Estonia	143	Kuwait	143	Peru	143
South Sudan	144	Singapore	144	Qatar	144	Chad	144
United Arab Emirates	145	Uruguay	145	Montenegro	145	Belize	145
Jamaica	146	Djibouti	146	Gabon	146	Burundi	146
Jordan	147	Fiji	147	Brunei	147	Liberia	147
Malawi	148	Equatorial Guinea	148	Botswana	148	Cameroon	148
Oman	149	Guyana	149	Gambia	149	Rwanda	149
Palestine	150	Montenegro	150	Barbados	150	Senegal	150
Mauritius	151	Suriname	151	Namibia	151	Guatemala	151
Malta	152	Bhutan	152	Guyana	152	Togo	152
Trinidad and Tobago	153	Maldives	153	Fiji	153	Bahamas	153
Kuwait	154	Cyprus	154	Bhutan	154	Haiti	154
Barbados	155	Bahamas	155	Bahrain	155	Niger	155
Namibia	156	Belize	156	Djibouti	156	Benin	156

Guyana	157	Barbados	157	Suriname	157	Eritrea	157
Bhutan	158	Luxembourg	158	Bahamas	158	Ecuador	158
Bahrain	159	Sao Tome and Principe	159	Belize	159	Barbados	159
Djibouti	160	Saint Lucia	160	Saint Lucia	160	Dominica	160
Bahamas	161	Brunei	161	Equatorial Guinea	161	Bhutan	161
Belize	162	Malta	162	Andorra	162	Ethiopia	162
Saint Lucia	163	Seychelles	163	Bermuda	163	Nigeria	163
Bermuda	164	Iceland	164	Sao Tome and Principe	164	Sao Tome and Principe	164
Sao Tome and Principe	165	Grenada	165	Dominica	165	Panama	165
Dominica	166	Antigua and Barbuda	166	Grenada	166	Ghana	166
Grenada	167	Dominica	167	Antigua and Barbuda	167	Equatorial Guinea	167
Antigua and Barbuda	168	Bermuda	168	Maldives	168	Antigua and Barbuda	168
Maldives	169	Andorra	169	Greenland	169		
Seychelles	170	Greenland	170	Seychelles	170		

Table S4. Country list corresponding to the index in the horizontal axis of Figure S1.

Alzhemers Dementia		Stomach Cancer		Liver Cancer		Lung Cancer	
**Country**	**Index**	**Country**	**Index**	**Country**	**Index**	**Country**	**Index**
Finland	1	Mongolia	1	Mongolia	1	Hungary	1
Kuwait	2	China	2	Laos	2	Serbia	2
Turkey	3	Kyrgyzstan	3	Viet Nam	3	Montenegro	3
Saudi Arabia	4	Albania	4	Egypt	4	China	4
United Kingdom	5	Guatemala	5	Gambia	5	Denmark	5
Tunisia	6	Bhutan	6	China	6	Poland	6
Libya	7	Ecuador	7	Thailand	7	Croatia	7
United States	8	Sao Tome	8	Cambodia	8	Netherlands	8
Syria	9	Kazakhstan	9	Guinea	9	Turkey	9
Lebanon	10	Russia	10	Liberia	10	Belgium	10
Bahrain	11	Costa Rica	11	Sierra Leone	11	Canada	11
Netherlands	12	Iran	12	Burkina Faso	12	Slovenia	12
Myanmar	13	Honduras	13	South Korea	13	Romania	13
Jordan	14	Belarus	14	Guatemala	14	Cuba	14
Iceland	15	Chile	15	Rwanda	15	United States	15
Morocco	16	El Salvador	16	Ghana	16	United Kingdom	16
Sweden	17	Japan	17	Nigeria	17	France	17
Switzerland	18	Colombia	18	Togo	18	Albania	18
Iran	19	Viet Nam	19	Mauritania	19	Uruguay	19
Yemen	20	Afghanistan	20	Guinea-Bissau	20	Greece	20
Norway	21	Peru	21	Philippines	21	Bulgaria	21
Canada	22	South Korea	22	Honduras	22	Bosnia/Herzeg.	22
Algeria	23	Lithuania	23	Myanmar	23	Russia	23
Greece	24	Turkey	24	Senegal	24	Slovakia	24
Sudan	25	Latvia	25	Nicaragua	25	Germany	25
France	26	Azerbaijan	26	Benin	26	Estonia	26
Belgium	27	Ukraine	27	Belize	27	Ireland	27
Australia	28	Estonia	28	Dominican Rep.	28	Czech Republic	28
Spain	29	Portugal	29	Singapore	29	Latvia	29
Denmark	30	Saint Lucia	30	Indonesia	30	Luxembourg	30
Ireland	31	Myanmar	31	Moldova	31	Norway	31
Egypt	32	Nicaragua	32	Congo	32	Brunei	32
Qatar	33	Panama	33	Japan	33	Spain	33
Nicaragua	34	Kenya	34	Romania	34	Viet Nam	34
Bosnia/Herzeg.	35	Romania	35	Qatar	35	Lithuania	35

Peru	36	Togo	36	Kyrgyzstan	36	Italy	36
Oman	37	Rwanda	37	Uganda	37	Timor-Leste	37
El Salvador	38	Uruguay	38	Fiji	38	Iceland	38
Iraq	39	Croatia	39	Brunei	39	Austria	39
New Zealand	40	Venezuela	40	El Salvador	40	South Korea	40
Montenegro	41	Moldova	41	Malaysia	41	Cyprus	41
Afghanistan	42	Mali	42	Grenada	42	Singapore	42
Paraguay	43	Bulgaria	43	Suriname	43	Kazakhstan	43
Malaysia	44	Uzbekistan	44	Bosnia/Herzeg.	44	Armenia	44
Poland	45	Armenia	45	Montenegro	45	Thailand	45
Gabon	46	Hungary	46	France	46	Mongolia	46
Maldives	47	Georgia	47	Chad	47	New Zealand	47
Haiti	48	Slovenia	48	Italy	48	Belarus	48
Georgia	49	Brazil	49	Armenia	49	Japan	49
Suriname	50	Slovakia	50	Haiti	50	Argentina	50
Belarus	51	Montenegro	51	Ecuador	51	Ukraine	51
Armenia	52	Haiti	52	Georgia	52	Switzerland	52
Thailand	53	Jamaica	53	Peru	53	Australia	53
Equ. Guinea	54	Italy	54	South Sudan	54	South Africa	54
Cuba	55	Serbia	55	Bulgaria	55	Malta	55
Kazakhstan	56	Belize	56	Kazakhstan	56	Portugal	56
Uruguay	57	Bosnia/Herzeg.	57	Albania	57	Israel	57
Bulgaria	58	Poland	58	Zimbabwe	58	Myanmar	58
Luxembourg	59	Dominican Rep.	59	Costa Rica	59	Malaysia	59
Slovakia	60	Brunei	60	Timor-Leste	60	Finland	60
Laos	61	Zimbabwe	61	Afghanistan	61	Moldova	61
Nepal	62	Grenada	62	Slovenia	62	Indonesia	62
Dominican Rep.	63	Syria	63	Luxembourg	63	Sweden	63
Viet Nam	64	Somalia	64	Mexico	64	Sao Tome	64
Barbados	65	Bolivia	65	Greece	65	Libya	65
Indonesia	66	Spain	66	Libya	66	Syria	66
Albania	67	Paraguay	67	Croatia	67	Philippines	67
China	68	Argentina	68	Niger	68	Georgia	68
Italy	69	Singapore	69	Kenya	69	Lebanon	69
Timor-Leste	70	Mauritius	70	South Africa	70	Venezuela	70
Azerbaijan	71	Mexico	71	Spain	71	Grenada	71
Seychelles	72	Nepal	72	Equ. Guinea	72	Tunisia	72
Angola	73	Germany	73	Saudi Arabia	73	Jordan	73

Bolivia	74	Senegal	74	Austria	74	Bahrain	74
Malawi	75	India	75	Burundi	75	Paraguay	75
Israel	76	Austria	76	Bhutan	76	Seychelles	76
Sri Lanka	77	Bahamas	77	Azerbaijan	77	Jamaica	77
Rwanda	78	Czech Republic	78	Portugal	78	Belize	78
Mauritania	79	Cyprus	79	Syria	79	Iraq	79
Gambia	80	Barbados	80	Colombia	80	Suriname	80
Mongolia	81	Israel	81	Serbia	81	Laos	81
Cambodia	82	Greece	82	Chile	82	Brazil	82
Argentina	83	South Sudan	83	Cameroon	83	Dominican Rep.	83
Portugal	84	Cambodia	84	Angola	84	Chile	84
Kenya	85	Qatar	85	Brazil	85	Cambodia	85
Germany	86	Bangladesh	86	Kuwait	86	Nepal	86
Bhutan	87	Madagascar	87	Slovakia	87	Morocco	87
Burundi	88	Iraq	88	Russia	88	Colombia	88
Mali	89	Uganda	89	Sudan	89	Kyrgyzstan	89
Honduras	90	Jordan	90	United States	90	Azerbaijan	90
Ghana	91	Algeria	91	Cyprus	91	Trinidad/Tob.	91
Benin	92	Seychelles	92	Seychelles	92	Qatar	92
Togo	93	Cuba	93	Mali	93	Bangladesh	93
Burkina Faso	94	Antigua/Bar.	94	Hungary	94	Mauritius	94
Chile	95	Sri Lanka	95	Gabon	95	Peru	95
Tanzania	96	Suriname	96	Iceland	96	Saint Lucia	96
Senegal	97	Malta	97	Switzerland	97	Panama	97
Uganda	98	Ireland	98	Panama	98	Algeria	98
Pakistan	99	South Africa	99	Uzbekistan	99	Iran	99
Mozambique	100	Luxembourg	100	Venezuela	100	Bahamas	100
Ethiopia	101	Oman	101	Mozambique	101	India	101
Djibouti	102	Burundi	102	Bahamas	102	Maldives	102
Hungary	103	Zambia	103	Jordan	103	Barbados	103
Zimbabwe	104	France	104	Iraq	104	Afghanistan	104
Namibia	105	Netherlands	105	Germany	105	Madagascar	105
Sierra Leone	106	New Zealand	106	Australia	106	Kuwait	106
Cameroon	107	Trinidad/Tob.	107	Belgium	107	Egypt	107
Nigeria	108	Bahrain	108	Cuba	108	Haiti	108
Niger	109	United Kingdom	109	Finland	109	Nicaragua	109
India	110	Denmark	110	Denmark	110	Ecuador	110
Congo	111	Finland	111	United Kingdom	111	Uzbekistan	111

Cyprus	112	Lebanon	112	Argentina	112	Costa Rica	112
Guinea-Bissau	113	Yemen	113	Czech Republic	113	Mexico	113
Liberia	114	Belgium	114	Guyana	114	Honduras	114
South Korea	115	Malaysia	115	Ireland	115	Bhutan	115
Chad	116	Angola	116	Somalia	116	Gabon	116
Botswana	117	Norway	117	Madagascar	117	El Salvador	117
South Africa	118	Libya	118	Eritrea	118	Sri Lanka	118
Malta	119	Morocco	119	Pakistan	119	Fiji	119
Czech Republic	120	Tunisia	120	Lithuania	120	Pakistan	120
Sao Tome	121	Liberia	121	Canada	121	Saudi Arabia	121
Eritrea	122	Maldives	122	Antigua/Bar.	122	Antigua/Bar.	122
South Sudan	123	Switzerland	123	Djibouti	123	Oman	123
Fiji	124	Iceland	124	Oman	124	Bolivia	124
Croatia	125	Mauritania	125	Latvia	125	Guyana	125
Zambia	126	Sierra Leone	126	Sweden	126	Equ. Guinea	126
Grenada	127	Guinea-Bissau	127	Israel	127	Guatemala	127
Guinea	128	Canada	128	Barbados	128	Botswana	128
Somalia	129	Australia	129	Paraguay	129	Zimbabwe	129
Austria	130	Burkina Faso	130	Estonia	130	Yemen	130
Brazil	131	Tanzania	131	Bolivia	131	Namibia	131
Brunei	132	Sweden	132	Trinidad/Tob.	132	Ethiopia	132
Madagascar	133	Ghana	133	Zambia	133	Djibouti	133
Bangladesh	134	Guyana	134	New Zealand	134	Somalia	134
Costa Rica	135	Saudi Arabia	135	Turkey	135	Mozambique	135
Serbia	136	Ethiopia	136	Poland	136	Eritrea	136
Bahamas	137	Indonesia	137	Mauritius	137	Kenya	137
Belize	138	Pakistan	138	Sri Lanka	138	Uganda	138
Trinidad/Tob.	139	Philippines	139	Botswana	139	Ghana	139
Japan	140	Guinea	140	Ukraine	140	Burkina Faso	140
Russia	141	Eritrea	141	Netherlands	141	Mali	141
Jamaica	142	Djibouti	142	Yemen	142	South Sudan	142
Panama	143	Thailand	143	Maldives	143	Gambia	143
Latvia	144	Congo	144	Saint Lucia	144	Angola	144
Antigua/Bar.	145	Benin	145	Malta	145	Sudan	145
Romania	146	Fiji	146	Iran	146	Rwanda	146
Lithuania	147	Malawi	147	Jamaica	147	Guinea-Bissau	147
Estonia	148	United States	148	India	148	Senegal	148
Ecuador	149	Cameroon	149	Belarus	149	Zambia	149

Slovenia	150	Egypt	150	Norway	150	Burundi	150
Mexico	151	Laos	151	Bahrain	151	Togo	151
Guatemala	152	Nigeria	152	Bangladesh	152	Sierra Leone	152
Moldova	153	Equ. Guinea	153	Uruguay	153	Cameroon	153
Guyana	154	Gabon	154	Lebanon	154	Liberia	154
Venezuela	155	Chad	155	Tanzania	155	Mauritania	155
Saint Lucia	156	Timor-Leste	156	Namibia	156	Congo	156
Ukraine	157	Sudan	157	Ethiopia	157	Chad	157
Colombia	158	Niger	158	Malawi	158	Guinea	158
Mauritius	159	Namibia	159	Sao Tome	159	Nigeria	159
Uzbekistan	160	Gambia	160	Algeria	160	Benin	160
Philippines	161	Kuwait	161	Morocco	161	Malawi	161
Kyrgyzstan	162	Mozambique	162	Tunisia	162	Tanzania	162
Singapore	163	Botswana	163	Nepal	163	Niger	163
**Leukemia**		**Kidney Disease**		**Inflammatory Heart Disease**		**Rheumatic Heart Disease**	
**Country**	**Index**	**Country**	**Index**	**Country**	**Index**	**Country**	**Index**
Kazakhstan	1	Nicaragua	1	Serbia	1	Pakistan	1
Iraq	2	El Salvador	2	Bosnia/Herzeg.	2	Bhutan	2
Syria	3	Fiji	3	Russia	3	India	3
Seychelles	4	Bolivia	4	Latvia	4	Burkina Faso	4
Saint Lucia	5	Seychelles	5	Egypt	5	Angola	5
Ecuador	6	Saudi Arabia	6	Montenegro	6	Fiji	6
Eritrea	7	Sao Tome	7	Poland	7	Nepal	7
Greece	8	Libya	8	Bulgaria	8	Haiti	8
Israel	9	Jordan	9	Belarus	9	Georgia	9
Ethiopia	10	Kazakhstan	10	Ukraine	10	Sierra Leone	10
Yemen	11	Guatemala	11	South Africa	11	Chad	11
Latvia	12	Yemen	12	Saint Lucia	12	Argentina	12
Colombia	13	Suriname	13	Fiji	13	Sao Tome	13
Egypt	14	Thailand	14	Albania	14	Mali	14
Djibouti	15	Egypt	15	Syria	15	Myanmar	15
France	16	Afghanistan	16	Ghana	16	Mongolia	16
Grenada	17	Philippines	17	Algeria	17	Equ. Guinea	17
Costa Rica	18	Laos	18	Botswana	18	Eritrea	18
Cyprus	19	Zimbabwe	19	Haiti	19	Madagascar	19
Hungary	20	Paraguay	20	Romania	20	China	20
Paraguay	21	Sierra Leone	21	Guyana	21	Laos	21
United States	22	Bhutan	22	Saudi Arabia	22	Benin	22

Iran	23	Haiti	23	Lithuania	23	Zimbabwe	23
Jordan	24	Kuwait	24	Equ. Guinea	24	Yemen	24
Croatia	25	Bahrain	25	Namibia	25	Togo	25
Italy	26	Tunisia	26	Angola	26	Gambia	26
Panama	27	Sudan	27	Estonia	27	Cameroon	27
Estonia	28	Peru	28	Argentina	28	Cambodia	28
Belgium	29	Iraq	29	Yemen	29	Burundi	29
Austria	30	Myanmar	30	Zimbabwe	30	Somalia	30
Slovakia	31	South Africa	31	Barbados	31	Nigeria	31
El Salvador	32	India	32	Sudan	32	Congo	32
Uruguay	33	Cambodia	33	Burkina Faso	33	Afghanistan	33
Somalia	34	Cameroon	34	Brazil	34	Uganda	34
Cambodia	35	Uzbekistan	35	Bahamas	35	Niger	35
New Zealand	36	Gambia	36	Myanmar	36	Mozambique	36
Lithuania	37	Guyana	37	Suriname	37	Guinea	37
Denmark	38	Belize	38	Hungary	38	Sudan	38
Australia	39	Sri Lanka	39	Moldova	39	Timor-Leste	39
Turkey	40	Ecuador	40	Kyrgyzstan	40	Kazakhstan	40
Viet Nam	41	Benin	41	Kazakhstan	41	Namibia	41
Slovenia	42	Algeria	42	Iraq	42	South Sudan	42
Canada	43	Nepal	43	Belize	43	Guinea-Bissau	43
Antigua/Bar.	44	Morocco	44	Libya	44	Kyrgyzstan	44
Luxembourg	45	Indonesia	45	Gabon	45	Ghana	45
Serbia	46	Maldives	46	Madagascar	46	Gabon	46
Germany	47	Mexico	47	Bolivia	47	South Africa	47
Sudan	48	Togo	48	Afghanistan	48	Senegal	48
Portugal	49	Dominican Rep.	49	Slovenia	49	Botswana	49
Netherlands	50	Mali	50	Sierra Leone	50	Ethiopia	50
Lebanon	51	Mauritania	51	Mongolia	51	Bolivia	51
Belarus	52	Turkey	52	Tunisia	52	Mauritania	52
Romania	53	Guinea-Bissau	53	Djibouti	53	Djibouti	53
Czech Republic	54	Argentina	54	Bahrain	54	Liberia	54
Libya	55	Chad	55	Morocco	55	Malawi	55
United Kingdom	56	Qatar	56	Austria	56	Rwanda	56
Philippines	57	Ghana	57	Greece	57	Tanzania	57
Fiji	58	Grenada	58	Cameroon	58	Belarus	58
Thailand	59	Equ. Guinea	59	Congo	59	Philippines	59
Guatemala	60	Timor-Leste	60	Nigeria	60	Iraq	60

Kenya	61	Liberia	61	Eritrea	61	Zambia	61
Trinidad/Tob.	62	Guinea	62	United States	62	Morocco	62
Jamaica	63	Botswana	63	Panama	63	Uzbekistan	63
Barbados	64	Trinidad/Tob.	64	Uruguay	64	Indonesia	64
South Sudan	65	Oman	65	Mozambique	65	Azerbaijan	65
Ireland	66	Senegal	66	Uganda	66	Armenia	66
Timor-Leste	67	Lebanon	67	Burundi	67	Egypt	67
Indonesia	68	Panama	68	Laos	68	Suriname	68
Sweden	69	Israel	69	Chad	69	Ukraine	69
Malaysia	70	Viet Nam	70	Trinidad/Tob.	70	Kenya	70
Spain	71	Angola	71	Mali	71	Bangladesh	71
Russia	72	Romania	72	Finland	72	Brunei	72
Suriname	73	Serbia	73	Gambia	73	Russia	73
Poland	74	Namibia	74	Benin	74	Sri Lanka	74
Chile	75	Mongolia	75	Lebanon	75	Bulgaria	75
Uganda	76	Bahamas	76	Rwanda	76	Maldives	76
Norway	77	Malaysia	77	South Sudan	77	Dominican Rep.	77
Albania	78	Niger	78	Togo	78	Croatia	78
Honduras	79	Barbados	79	Brunei	79	Saint Lucia	79
Switzerland	80	Bangladesh	80	Tanzania	80	New Zealand	80
Oman	81	Pakistan	81	Slovakia	81	Algeria	81
Montenegro	82	Uruguay	82	Malaysia	82	Slovenia	82
Brazil	83	Nigeria	83	Sao Tome	83	Poland	83
Madagascar	84	Iran	84	Ethiopia	84	Malaysia	84
Nicaragua	85	Burkina Faso	85	Cambodia	85	Luxembourg	85
Cuba	86	Colombia	86	Indonesia	86	Viet Nam	86
Mexico	87	Kyrgyzstan	87	Switzerland	87	Peru	87
Myanmar	88	Saint Lucia	88	Croatia	88	Cyprus	88
China	89	Djibouti	89	Somalia	89	Seychelles	89
Laos	90	Azerbaijan	90	Cuba	90	Hungary	90
Dominican Rep.	91	Mauritius	91	Mauritius	91	Israel	91
Mozambique	92	Costa Rica	92	Thailand	92	Spain	92
South Africa	93	Venezuela	93	Mauritania	93	Lithuania	93
Argentina	94	Cyprus	94	Spain	94	Libya	94
Peru	95	Brazil	95	Costa Rica	95	Tunisia	95
Venezuela	96	Georgia	96	Germany	96	Italy	96
Algeria	97	China	97	Georgia	97	Czech Republic	97
Sri Lanka	98	Gabon	98	Malawi	98	Montenegro	98

Ukraine	99	Jamaica	99	Zambia	99	Germany	99
Nepal	100	United States	100	Guinea-Bissau	100	Paraguay	100
Armenia	101	Estonia	101	Sri Lanka	101	Moldova	101
Qatar	102	Chile	102	Grenada	102	Saudi Arabia	102
Bulgaria	103	Bosnia/Herzeg.	103	Oman	103	Belize	103
Mauritius	104	Greece	104	Jamaica	104	Oman	104
Sao Tome	105	Montenegro	105	Guinea	105	Latvia	105
Bhutan	106	Bulgaria	106	Senegal	106	France	106
Finland	107	Honduras	107	Timor-Leste	107	Albania	107
Kuwait	108	Antigua/Bar.	108	Iran	108	Belgium	108
Moldova	109	Brunei	109	Kuwait	109	Austria	109
Belize	110	Singapore	110	Belgium	110	Brazil	110
Saudi Arabia	111	Mozambique	111	Kenya	111	Guyana	111
Iceland	112	South Sudan	112	Turkey	112	Trinidad/Tob.	112
Tunisia	113	Germany	113	Philippines	113	Costa Rica	113
Japan	114	Belgium	114	Liberia	114	Slovakia	114
Kyrgyzstan	115	Uganda	115	France	115	Kuwait	115
Georgia	116	Moldova	116	Paraguay	116	Bosnia/Herzeg.	116
Pakistan	117	Croatia	117	Dominican Rep.	117	Ecuador	117
Afghanistan	118	Burundi	118	Viet Nam	118	Romania	118
Guyana	119	Japan	119	New Zealand	119	Syria	119
Bolivia	120	Ethiopia	120	Seychelles	120	Grenada	120
Uzbekistan	121	Congo	121	Netherlands	121	Jamaica	121
Rwanda	122	Slovakia	122	Australia	122	Nicaragua	122
Malta	123	Somalia	123	Azerbaijan	123	Lebanon	123
Morocco	124	Syria	124	Singapore	124	Australia	124
Azerbaijan	125	Italy	125	Niger	125	Cuba	125
South Korea	126	Austria	126	Czech Republic	126	Estonia	126
Singapore	127	Albania	127	Cyprus	127	Greece	127
Burundi	128	South Korea	128	Chile	128	Bahrain	128
Bahrain	129	Eritrea	129	Luxembourg	129	Portugal	129
Chad	130	Rwanda	130	Italy	130	Jordan	130
Congo	131	Poland	131	Honduras	131	Mexico	131
India	132	Tanzania	132	Peru	132	United Kingdom	132
Haiti	133	Latvia	133	Ireland	133	El Salvador	133
Bosnia/Herzeg.	134	Malawi	134	Portugal	134	Thailand	134
Bahamas	135	Spain	135	Sweden	135	Ireland	135
Zimbabwe	136	Zambia	136	Colombia	136	Bahamas	136

Cameroon	137	Armenia	137	Nicaragua	137	Uruguay	137
Angola	138	Portugal	138	Maldives	138	Netherlands	138
Nigeria	139	Ireland	139	Antigua/Bar.	139	Japan	139
Mongolia	140	Kenya	140	Jordan	140	Canada	140
Zambia	141	Luxembourg	141	United Kingdom	141	Denmark	141
Namibia	142	Hungary	142	Venezuela	142	United States	142
Bangladesh	143	Netherlands	143	Denmark	143	Sweden	143
Maldives	144	Australia	144	Japan	144	Chile	144
Gabon	145	Malta	145	Norway	145	Iran	145
Guinea	146	Czech Republic	146	El Salvador	146	Qatar	146
Burkina Faso	147	Cuba	147	Canada	147	Colombia	147
Togo	148	Slovenia	148	China	148	Turkey	148
Tanzania	149	Canada	149	Malta	149	Norway	149
Niger	150	France	150	Ecuador	150	Panama	150
Ghana	151	Norway	151	Bhutan	151	Mauritius	151
Botswana	152	Switzerland	152	Iceland	152	Venezuela	152
Senegal	153	Lithuania	153	Israel	153	Serbia	153
Benin	154	Iceland	154	Qatar	154	Honduras	154
Equ. Guinea	155	Madagascar	155	Uzbekistan	155	Singapore	155
Liberia	156	Russia	156	Armenia	156	Switzerland	156
Mauritania	157	Denmark	157	South Korea	157	Malta	157
Sierra Leone	158	Sweden	158	Pakistan	158	Iceland	158
Mali	159	Belarus	159	Mexico	159	South Korea	159
Brunei	160	New Zealand	160	Guatemala	160	Finland	160
Malawi	161	Ukraine	161	India	161	Barbados	161
Guinea-Bissau	162	United Kingdom	162	Nepal	162	Guatemala	162
Gambia	163	Finland	163	Bangladesh	163	Antigua/Bar.	163
